# Standardised Versioning of Datasets: a FAIR–compliant Proposal

**DOI:** 10.1038/s41597-024-03153-y

**Published:** 2024-04-09

**Authors:** Alba González–Cebrián, Michael Bradford, Adriana E. Chis, Horacio González–Vélez

**Affiliations:** Cloud Competency Centre, National College of Ireland, Dublin, Ireland

**Keywords:** Research data, Research management, Technology

## Abstract

This paper presents a standardised dataset versioning framework for improved reusability, recognition and data version tracking, facilitating comparisons and informed decision-making for data usability and workflow integration. The framework adopts a software engineering-like data versioning nomenclature (“major.minor.patch”) and incorporates data schema principles to promote reproducibility and collaboration. To quantify changes in statistical properties over time, the concept of *data drift metrics* (*d*) is introduced. Three metrics (*d*_*P*_, *d*_*E*_,_*PCA*_, and *d*_*E,AE*_) based on unsupervised Machine Learning techniques (Principal Component Analysis and Autoencoders) are evaluated for dataset creation, update, and deletion. The optimal choice is the *d*_*E*_,_*PCA*_ metric, combining PCA models with splines. It exhibits efficient computational time, with values below 50 for new dataset batches and values consistent with seasonal or trend variations. Major updates (i.e., values of 100) occur when scaling transformations are applied to over 30% of variables while efficiently handling information loss, yielding values close to 0. This metric achieved a favourable trade-off between interpretability, robustness against information loss, and computation time.

## Introduction

The significance of data versioning lies in its capacity to monitor changes over time, promote reproducibility, and encourage collaboration. While the Research Data Alliance (RDA) and the DataCite Metadata Schema^1,2^ incorporate versioning for data citation, their frameworks have limitations in aligning with the FAIR (Findable, Accessible, Interoperable, and Reusable) principles^3^. For instance, they often rely on traditional publication indexing, hindering efficient discovery based on dataset attributes. Inadequate tools for understanding version differences led to users navigating dense documentation, resulting in inefficient discovery, low reusability of already preprocessed datasets and sub-optimal resource reuse, undermining the recognition of previous data curation efforts and, ultimately, reproducibility^4^.

To address these issues, we propose optimising the DataCite schema to enhance the findability, reusability, and recognition of data versions. A set of foundational principles for effective data versioning practices^5^ emphasises the need to identify new versions, distinguish between revisions, releases, granularity levels, and dataset manifestations, and comply with provenance and citation standards. While some research has explored data monitoring or timestamp-based data versioning, a clear standard that encompasses and reflects the multiple dimensions of changes in data versions is lacking.

In this study, we present two layers of the same global solution. Firstly, we propose a standard data version tag based on the widely recognised software versioning format of “major.minor.patch”. This aligns with the foundational principles from Klump *et al*.^5^ and complies with existing fields in the DataCite schema. Secondly, we introduce the concept of a *data drift field* to be integrated into the DataCite metadata schema, serving as the “minor” term of the version tag. We introduce three data drift metrics leveraging Machine Learning (ML) models to capture and quantify data drift, i.e., changes in statistical properties or distributions across data versions, namely *d*_*P*_ using Principal Component Analysis (PCA), *d*_*E,PCA*_ using PCA and splines, and *d*_*E,AE*_ using Autoencoders (AE) and splines.

Our experimental approach focused on working with real datasets to reflect real-world data dynamics. The methodology involves performing common operations, including the creation of new information, updates to existing records, and deletions, simulating scenarios frequently encountered in dynamic datasets. This systematic approach serves to validate the technical quality of our dataset while introducing common changes that often occur in real-world data. Therefore, our contribution lies not only in proposing a standard data versioning nomenclature but also in demonstrating the practical value of this approach by applying it to real datasets.

We outline the structure of the paper as follows: Section 2.1 describes the proposed data versioning nomenclature and explores potential metrics to quantify the “minor” term, specifically related to data drift. The methods used to calculate the three metrics mentioned above are further explained in Section 2.2. Section 2.4 describes the seven open datasets used to evaluate this work, including their source repository and relevant features. Section 3 presents the results, which are further discussed from Sections 4.1 to 4.3, demonstrating the coherence of the proposed metrics across different datasets and scenarios and highlighting the advantages and disadvantages of each metric. Both *d*_*E,PCA*_ and *d*_*E,AE*_ metrics exhibited similar behaviours, indicating higher data drift values for atypical data batches and variable scale updates. The *d*_*P*_ metric showed less sensitivity to scale changes, given that the covariance structure remained unaffected. Simulated information loss demonstrated that *d*_*E,PCA*_ was more stable than *d*_*P*_ in the data drift values, preserving recognition of records in the reference dataset. The AE-based metric, *d*_*E,AE*_, displayed higher data drift values for noisy datasets, implying a need to refine the model-building stage. Regarding computational resources, *d*_*E,PCA*_ had an intermediate model-building time and remained the fastest technique in execution time for revisions across all datasets and experiments. Overall, the *d*_*E,PCA*_ metric offers the optimal stability, robustness, and computation time trade-off. Furthermore, the results highlighted the impact of dataset characteristics, suggesting potential contributions for specific data attributes. Lastly, Section 4.4 summarises the paper’s main ideas and contributions, including proposing a standardised data version labelling based on existing DataCite fields while allowing for domain-specific solutions within the standard.

## Methods

In this section, first, we present in Section 2.1 a conceptual and technical definition of the proposed standard for naming data versions. Next, in Section 2.2 we describe different approaches to determine a standard minor term, specifically a data drift metric. Three different data drift metrics (*d*) will be proposed, being based either on the PCA loadings (***P***, i.e., *d*_*P*_) or on the reconstruction error (***E***) obtained after projecting the data matrix onto the model ${\mathscr{M}}$ (i.e., ${d}_{E,{\mathscr{M}}}$), which can be a PCA (*d*_*E,PCA*_) or an Autoencoder model (*d*_*E,AE*_). Lastly, we describe the experimental setup implemented to assess these approaches. Throughout this paper, the term “revisions” refers to new versions of a dataset, following the terminology proposed by Klump *et al*.^5^.

### The proposed solution

In line with Klump *et al*.‘s Foundational Principle #6, our proposed versioning protocol aims to adopt the traditional versioning semantics used in software, which follows the “major.minor.patch” format. However, universality and modularity must be considered to establish a standardised data versioning nomenclature based on this syntax. Universality refers to the ability to work with common concepts across various contexts and industries, avoiding the creation of *ad hoc* version naming systems for each field or environment. Modularity entails employing strategies based on orthogonal components or modules that can operate independently of one another. This provides flexibility in determining the technical tools used to quantify each term of the version tag, which may vary depending on the specific context or environment. Taking these requirements into account, we propose the following naming standard for data versions:

1. *Patch Version* (“*major.minor.patch*”): In data versioning, a patch version update typically involves small, specific fixes or corrections applied to the dataset. These updates address inconsistencies, errors, or bugs without significantly changing the data structure or functionality. Patch updates are generally backwards-compatible, allowing the seamless application to existing datasets without major modifications. We propose representing the patch version with timestamps that identify specific operations. The DataCite Metadata Schema includes the “datacite.VersionDate” field, dedicated to capturing timestamping information related to data versions in a precise and standardised manner^2^. This field adheres to recognised timestamp formats like ISO 8601, ensuring consistent representation and interoperability across datasets. It enables accurate documentation and tracking of temporal changes in dataset versions, facilitating navigation and interpretation based on temporal characteristics within the academic and research community.
2. *Minor Version* (“major.minor.patch”): A minor version update in data versioning typically encompasses enhancements, additions, or updates that do not significantly disrupt the existing data structure or compatibility and may improve data processing mechanisms. This information can be represented by a data drift metric, quantifying the informational change between a data version and a reference point, typically the oldest version sharing the same data model (major term). Standardised data drift metrics must be defined and agreed upon to capture the extent of drift between dataset versions, a topic explored in more detail in Subsection 2.2. The DataCite Metadata Schema’s “relatedIdentifier” element allows for specifying relationships between different dataset versions^2^. We propose incorporating the data drift metric as a custom metadata field, *“dataDriftMetric”*, within this element. This enables researchers to document and compare data drift metrics explicitly, providing insights into version changes, assessing reliability and relevance, and making informed decisions regarding data usability. Data consumers can better understand the implications of using specific dataset versions and evaluate the impact of data drift on downstream analyses.
3. *Major Version* (“*major.minor.patch*”): A major version update in data versioning signifies significant and substantial changes to the dataset, often involving substantial modifications, additions, and deletions to the data structure, schema, or underlying data model. Informing others about the data model or schema promotes data interoperability, understanding, and reuse. The DataCite Metadata Schema incorporates the “datacite.SchemaVersion” field to describe a dataset’s data model or schema in a comprehensive and structured manner^2^. This field allows the representation of commonly used data models or schemas, such as Dublin Core and Data Documentation Initiative (DDI). It also supports supplementary metadata fields such as “datacite.DataModelDescription” and “datacite.DataModelIdentifier”, which provide additional granularity regarding the data model. These fields encompass descriptions, identifiers, or controlled vocabularies, offering specific information about the structure, ontologies, or standards employed to define the dataset data model. By including the major version term, researchers can effectively communicate the underlying structural organisation and semantic relationships within the dataset, enabling others to interpret and integrate the data into their workflows and analyses while adhering to established data modelling practices and conventions.

The structure of the version label presented here mirrors the granularity levels used in traditional software versioning. It employs terms that embody general concepts applicable to all types of datasets, making it compliant with the requirement of universality and suitable for an interdisciplinary standard. Furthermore, it satisfies the modularity requirement since even if certain terms are updated (e.g., changing the technique for computing data drift or adjusting the level of time precision in timestamps), the conceptual meaning of the term within the version name remains consistent. The only requirement is maintaining coherence in the technical details applied across the versioning process to obtain the version terms.

Using the combination $< {\rm{minor,}}\,{\rm{patch}} > = < {\rm{data}}{\rm{.drift,}}\,{\rm{time}}{\rm{.stamp}} >$, it becomes efficient to map new versions regarding content drift and novelty while referencing the dataset source. Similarly, as new concepts are added, $< major,minor > = < data.model,data.drift >$ can trigger the reassessment of ML models based on previous data models. The minor term can serve as a standard metric for monitoring data across a data model, which is particularly beneficial for dynamic datasets. Subsection 2.2.1 describes different ML models used to compute the indices employed as the minor term, elaborated upon in Subsection 2.2.2.

### Standard data drift metrics for the minor term

This section presents various methodologies for quantifying data drift and integrating it as a fundamental component within a standardised content-based data versioning framework. The objective is to develop a universally applicable tool that enables the characterisation of content changes across different versions of data that adhere to the same data schema. Data drift is the concept which refers to changes or deviations that occur in the statistical properties or distributions of data over time. By quantifying and including data drift metrics within the metadata, researchers and data consumers can gain insights into the extent of changes, facilitating accurate comparisons between versions and making informed decisions regarding the suitability and reliability of different dataset versions.

There are several options in the literature for measuring data drift. Most basic methods are based on commands for comparing flat datasets, such as the “diff” Unix one. Yet, while such commands serve for dataset content comparison, they present several limitations, especially when dealing with complex or non-structured data. As they do not fit any model capturing the data structure, they might not be robust against changes in data formats, missing values, and other anomalies, making them less resilient to noisy or messy data, which are precisely very susceptible to yielding future data versions where some preprocessing or data cleaning was applied. Tools that look into the statistical properties of datasets might identify patterns invisible to simple character-by-character comparisons. A simple case could be the contention of a new data version by changing the numerical precision, which could lead to substantial data drift values using text-based comparison, whereas this information loss, as far as it does not affect the data patterns, would not yield an increase in data drift values based on statistical properties.

Some examples of classical statistical concepts for distance measurement are based on parametric approaches, such as the Kullback-Leibler (KL) divergence^6^ and the Jensen-Shannon Divergence (JSD)^7^, which measure the dissimilarity between the probability distributions of two datasets. However, these approaches are univariate, and applying them to multivariate datasets would require corrections to control high false positive rates^8^ when comparing the distances between dataset versions, which present their limitations themselves^9^. To overcome this limitation, multivariate approaches can be used instead for detecting changes in multivariate datasets.

Some examples of more sophisticated parametric techniques included the Cumulative Sum (CUSUM) algorithm^10^ and Bayesian Change Point Detection^11^, which compared the expected behaviour of the data with the observed new incoming values. However, recent approaches increasingly advocate using ML models for data monitoring^12^. These ML-based approaches leverage ensemble models^13^, the *k*-Nearest Neighbours (KNN) algorithm^14^, or autoencoders^15^ to detect and quantify data drift. These methods often redefine the quantification of data drift as a supervised problem, focusing on distinguishing between normal observations and atypical ones.

We propose using metrics derived from unsupervised ML algorithms to overcome the limitations of supervised approaches and the need for predefined categorisations. This approach enables the detection of changes in datasets regardless of their specific use for modelling tasks. However, it should be noted that unsupervised models do not directly capture the interoperability between data and the models based on them, such as changes in predictive performance. Assessing this aspect would require a generic data version tag representing the impact of new data versions on models, which is beyond the scope of data monitoring and falls under Continual Learning.

**Fig. 1:**
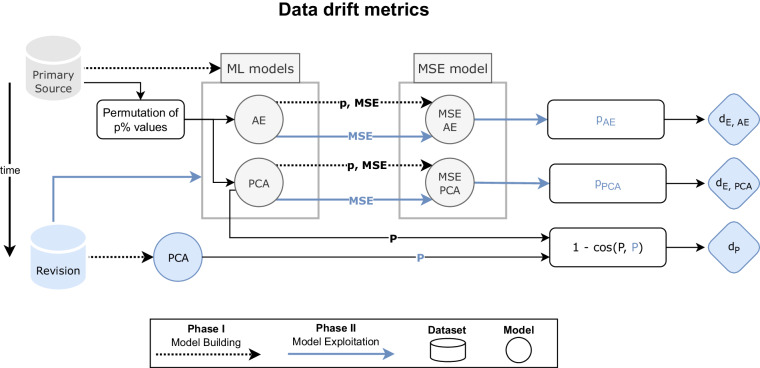
Flowchart summarising our approach to quantify data drift using several strategies. Each strategy employs an ML model and an associated data drift metric. First, for a Primary Source dataset, we build ML models and predictive models based on the Mean Squared Error (MSE models), conforming the Model Building Phase. Similarly, for a Revision dataset, i.e., a new dataset version, corresponding ML models are built. These models are used during the Model Exploitation Phase to compute the associated data drift metric (e.g., *d*_*E,AE*_, *d*_*E,PCA*_, and *d*_*P*_).

Figure 1 summarises our approach implemented to evaluate several strategies for quantifying data drift, all of which involve the combination of an ML model and a formula to compute a data drift index. All the models are built using the Primary Source dataset (Model Building flow in Fig. 1). Afterwards, they are used to compare the information with the new dataset versions, also called Revisions (Model Exploitation flow in Fig. 1), resulting in a certain data drift metric. In the Results section, we illustrate the behaviour of the proposed metric when different operations are applied to a Primary Source dataset to obtain new versions. However, it is important to note that other techniques for measuring data drift may be more suitable for specific datasets. The proposed framework is compatible with using alternative data drift metrics according to particular data models as long as they retain the same conceptual meaning within the proposed versioning nomenclature. Further research could explore different data drift metrics and their applicability to various data models. The following sections explain each proposed data drift metric used to compute the version numbers.

#### Unsupervised ML models

Unsupervised learning is the set of tools from ML used to extract information from the data without defining any explicit prediction problem. Among these approaches, some compression models aim to use a reduced-dimensionality representation of the data to reconstruct it. The common denominator of data compression models is that they trade lower precision of the reconstructed data for more compression power, i.e., by achieving a significant reduction of the dimensionality spanned by the dataset. In some of our previous work, we explored the use of PCA and AE to detect similarities across data versions^16^:

- **PCA:** Let ***X*** be a matrix with *N* observations on *K* variables. After some pre-processing, such as mean-centring and unit variance scaling, a PCA model is estimated^17^. This is done by compressing the ***X*** matrix into a subspace of lower dimension *A* (with *A* ≤ *K*). PCA is based on the bi-linear decomposition of ***X*** in ***X*** **=** ***TP***^⊤^ +***E***, where ***T*** is an *N* × *A* matrix of *scores* and ***P*** is a *K* × *A* matrix of *loadings*. The *A* columns of the loading matrix ***P*** are the *loading* vectors ***p***_*a*_, with $a=1,2,\ldots ,A$. The *score* matrix ***T*** can be considered as a collection of column vectors ***t***_*a*_ (latent variables or principal components), obtained as ***T*** = ***XP***, and with each one of them obtained as ${{\boldsymbol{t}}}_{a}={{\boldsymbol{Xp}}}_{a}$. From the scores matrix, one can recall the explained part of ***X*** in the PCA model as $\widehat{{\boldsymbol{X}}}=T{P}^{\top }$. Thus, the original data can be decomposed by the part explained (i.e., predicted) by the model (signal or $\widehat{{\boldsymbol{X}}}$), and the error not considered in any of the *A* latent variables (noise or ***E***). PCA models have also been proposed for data monitoring and implementing tools for Statistical process control (SPC), such as control charts to monitor the statistical properties of incoming data and detect shifts or abnormalities^18^.
- **AE:** It is a specific type of feed-forward neural network that involves a two-step process: encoding and decoding^19–21^. In this framework, the input data is compressed into a lower-dimensional code through the encoding function (*ϕ*: ***X*** → ***T***) and then reconstructed to obtain the output. The main purpose of AE is to perform dimensionality reduction, where the mathematical framework allows for the inclusion of non-linearities in the encoding and decoding process. The encoding function *ϕ* maps the observations in the input matrix ***X*** to a lower-dimensional representation ***T***. Subsequently, the decoding function $\psi$ reconstructs the low-dimensional representation ***T*** back into an approximation of the original input, denoted as $\widehat{{\boldsymbol{X}}}$. The goal of AE is to find a set of weights that minimise the reconstruction error, which is computed as the difference between the reconstructed output $\widehat{{\boldsymbol{X}}}$ and the original input ***X***, i.e., ${\boldsymbol{E}}=\widehat{{\boldsymbol{X}}}-{\boldsymbol{X}}$. AE aim to minimise this reconstruction error by optimising the weights, thereby obtaining a faithful representation of the original input. AE provide a powerful tool for learning meaningful representations of data by capturing relevant features in the lower-dimensional code ***T***. Including non-linear activation functions allows AE to capture complex patterns and relationships in the data, enabling them to perform efficient dimensionality reduction and reconstruction tasks.

#### Data drift indices for the minor term

From unsupervised models, two different indices are computed to quantify the data drift:

- **P-drift:** This metric can be used only if the PCA model is applied. The proposed metric to measure the drift between the Primary Source (PS, original version) and a Revision (R, newer version) of a dataset (*d*_*P*_) relies on the cosine of the angle between pairs of homologous loading vectors^22^. First, a PCA model for the ***X***^(*PS*)^ dataset is fitted, yielding the set of parameters $\{{{\boldsymbol{\lambda }}}^{(PS)},{{\boldsymbol{P}}}^{(PS)}\}$, where the *a* terms in the vector **λ** refer to the variances of each of the latent variables ***t***_*a*_. Next, a PCA model for the *R* dataset, yielding the same parameters: $\{{{\boldsymbol{\lambda }}}^{(R)},{{\boldsymbol{P}}}^{(R)}\}$. Both models are compared by computing the absolute value of the cosine between pairs of homologous loading vectors, and then a weighted sum is computed with all of them, as expressed in Eq. (1):
  1 $${d}_{P}=100\cdot \left(1-\frac{\sum _{a}{\lambda }_{a}| cos({{\boldsymbol{p}}}_{a}^{(PS)},{{\boldsymbol{p}}}_{a}^{(R)})| }{\sum _{a}{\lambda }_{a}}\right)\quad a\in 1,\ldots ,A$$
  These products are then weighted by the relative importance of their respective PC, added, and scaled to a range between 0 and 100. The relative importance is represented by the fraction of variance in the *a*-th PC.
- **E-drift:** On the other hand, this metric expressed in Eq. (2) quantifies the level of corruption or deviation in a dataset compared to a reference dataset. The E-drift is model-agnostic and rooted in the reconstruction Mean Squared Error (MSE), making it applicable regardless of the modelling technique. A permutation strategy is used to compute the E-drift. Different percentages of cells from the PS dataset are permuted, ranging from 1% to 100% with increments of 10%. These permutations are repeated *J* times (by default *J* = 10) to ensure that the model will not be based on a single random iteration of the process. The permuted datasets are projected onto the models fitted with the PS dataset, and the MSE is computed for each permutation percentage. These values of MSE and the percentage of permuted values are used to fit splines predicting the percentage of permuted (i.e., corrupted) values for a given MSE value. Splines are a mathematical technique that provides a smooth curve or surface to a set of data points^23^. The key notion is to try to fit the overall shape of a certain curve or surface as closely as possible by breaking it up into smaller segments, called “splines”, and then use mathematical functions, commonly cubic polynomials, to describe the shape of each spline. Splines can provide a smooth and continuous representation of the data, making them particularly useful when working with noisy data or describing curves with unknown generic shapes, as in this work.

The final expression used to obtain the E-drift value using the model ${\mathscr{M}}$, and having a fold of *J* splines obtained by doing *J* repetitions of each permutation level, is outlined in Eq. (2). The new dataset is passed through PCA and AE models, which reconstruct the data and yield the $MS{E}^{R,PCA}$ and $MS{E}^{R,AE}$ values, respectively. The percentage of permuted values (i.e.${p}_{j}^{{\mathscr{M}}}$) is estimated by the *J* splines fitted with the MSE values obtained by reconstructing permuted versions of the PS dataset, as explained above.

2 $${d}_{E,{\mathscr{M}}}=100\cdot \frac{\sum _{j}{p}_{j}^{{\mathscr{M}}}}{J}=100\cdot \frac{\sum _{j}{f}_{j}^{{\mathscr{M}}}(MS{E}^{R,{\mathscr{M}}})}{J}\quad i\in 1,\ldots ,J;\quad {\mathscr{M}}:AE,PCA$$

This process yields two values of E-drift: *d*_*E,PCA*_ and ${d}_{E,AE}$, corresponding to the MSE values and the splines obtained using the PCA and AE models, respectively. When the Revision dataset comes, it will be reconstructed by either the AE or the PCA model. This will yield a new value of the $MS{E}^{R,{\mathscr{M}}}$ passed to a fold of *J* splines ${\{{f}^{{\mathscr{M}}}\}}_{J}$. Each *j* spline will yield a certain value ${p}_{j}^{{\mathscr{M}}}$, equivalent to a percentage of permutation *p*(%) simulated on the Primary Source dataset. Finally, the result of the *J* splines will be averaged and scaled to a range between 0 and 100.

### Experimental setup

This work proposes a generic data drift metric based on ML models. To assess the behaviour of the metric with different datasets, several versioning events were tested, including creation, updates, and deletions:

- For the creation event, new information was added to the dataset, simulating dynamic datasets. In datasets already containing subsets, such as dataset DS 01, with data recorded in different months, the subsets were used to obtain the primary source and the block of new batches. The primary source was obtained for datasets without such partitions using the first 75% of the dataset. Sets of new observations were created by overlapping windows, with each iteration excluding the first record from the previous iteration and including the latest record. This allowed for assessing the coherence of the data drift indices within and between metrics. Moreover, the different time resolutions expressed by different sizes of the newly added batches also enabled us to explore the relationship between the changes in data drift metrics and the sample size of the Revisions.
- The update event involved changing existing records in the dataset and generating a new view. For these experiments, variables were transformed into a different scale. This was done by performing cubic root transformations on various percentages of columns. One would expect variable scale shifts to yield higher data drift values as the percentage of shifted variables increases. Substantial data drift values resulting from a change in scale could be interpreted as indicative of a change in the data model, particularly if the magnitudes of recordings of certain variables have undergone modifications. In such cases, elevated minor terms in the version tag should trigger major updates to the data version. In this scenario, looking at the *d*_*PCA*_ metric will be particularly interesting. Since, for its computation, a new PCA model is fitted on the Revision dataset, it could be insensitive to shifts in scale as far as they would not break the correlation structure between variables.
- The deletion event involved the removal of records present in the primary source dataset. To perform such experiments, a certain percentage of records was deleted from the Primary Source dataset by decimating the time series, simulating a lower sampling frequency. These deletion scenarios assessed how each unsupervised model could recognise the same patterns as in the Primary Source and how the information loss affected each data drift metric.

For each scenario, up to 100 repetitions were executed, changing the records within the added batches, the columns transformed, or the cells deleted for missing data imputation. For creation event experiments, the number of repetitions was limited by the number of samples of each memory size that could be obtained from the Revision dataset, e.g., for the batch size of 100%, only the whole Revision could be considered, without the possibility of changing the observations across repetitions. This was done to assess the repeatability of each data drift metric when the observations within the revision set varied.

Regarding pre-processing, the datasets were standardised using mean centering and unit variance scaling. PCA models were fitted to explain over 90% of the dataset variance. AEs for the primary source were trained by adding white noise to the original dataset to prevent over-fitting. The data generated following a white noise distribution was multiplied by a noise factor parameter, denoted as ${c}_{\varepsilon }$, which was used to adjust the noise level. Besides, 20 epochs were typically enough to ensure convergence of the AE. Finally, it is important to mention that the hyperparameters considered to optimise AE’s architecture included one or two hidden layers, with varying nodes within each layer, following previous works that used AEs to track changes over data^15^. The activation functions used were “relu” and “tanh” for the hidden layers and linear activation for the last layer.

The posterior analysis of the results obtained by the data drift metrics was coupled with the assessment provided by an exploratory analysis of data fluctuations over time. Such exploratory analysis was based on classical time series decomposition, treating each variable as the combination of a trend, seasonal and irregular component^24,25^. The technical details of this exploratory analysis and the results are in Section 3 of the Supplementary Material (Supplementary Figures 6 to 12). These insights were useful to determine if, for instance, the creation experiments yielding high data drift values corresponded to datasets where a trend was present. Besides, it also enabled us to check that the considered datasets covered different possibilities, such as short-term (i.e., daily) and long-term (i.e., yearly) seasonality.

### Open Dataset Selection and Description

The proposed versioning schema was evaluated using several time series datasets. Three key criteria guided the selection of datasets in this study. Firstly, the datasets were chosen for their openness, ensuring they were publicly accessible for transparency and reproducibility. This allowed users to scrutinise the production details and readily access the data to test the proposed data versioning standard. Secondly, the selected datasets contained dynamic information, particularly time series data, which undergo frequent changes such as updates, additions, and deletions. This dynamic nature was essential for evaluating the effectiveness of versioning tools. Lastly, the datasets were required to exhibit heterogeneity in terms of their temporal characteristics, encompassing trends and seasonality. This diversity in temporal features enabled the exploration of how the proposed data drift metrics responded to different changes across data versions. Nevertheless, the proposed methodology can be extended with all sorts of quantitative data sets, with AEs admitting categorical variables and PCA having some adaptations to deal with them^26^. Table 1 summarises the datasets used in this work. All of these datasets show a *long* dataset structure with more records than variables, which is typical in time series datasets. To provide further insights into the different dynamics of each dataset, a univariate exploratory analysis was carried out to assess the existence of trend and seasonality components (see Section 3 of the Supplementary Material for more information). Next, we provide a concise description of each dataset together with the rationale for its selection in our evaluation:

**Table 1:** Summary of the datasets used in the experiments assessing the proposed data drift metrics.

Identifier	Dataset	Records (*N*)	Variables (*K*)	*N*^(*PS*)^	Magnitudes	Trend	Seasonality
DS 01	SML2010^31^	4,137	22	2,764	continuous	no	daily
DS 02	Hungarian chickenpox cases^32^	522	20	262	counts	no	yearly
DS 03	Global land temperature^33^	1,365	485	1,200	continuous	yes	yearly
DS 04	Sales prediction^34^	64	4	48	continuous	yes	yearly
DS 05	Air quality^35^	9,357	12	7,110	continuous	no	daily
DS 06	Ozone level detection^36^	2,536	71	1,451	continuous	no	yearly
DS 07	Dublin footfall counts 2022^37^	8,760	99	6,569	counts	yes	weekly

*DS 01* –SML2010 dataset: This dataset consists of approximately 40 days of monitoring in a domotic house. It was used to fit a predictive module based on artificial neural networks for short-term forecasts of indoor temperature. The dataset contains two subsets: one captured during March and April 2011 (approximately 28 days) and the other captured in May 2011 (approximately 14 days). In total, 4,137 time instants are available. For experiments involving the addition of rows, the data from March were used as the Primary Source, and the batches from June were added in different sizes. This is an example of a dataset with a partition into two versions known *a priori*, which incorporated variables recording different magnitudes and presented a certain stationary component.

*DS 02* –Hungarian Chickenpox Cases dataset: This dataset consists of a time series of reported cases of chickenpox at the county level between 2005 and 2015. The dataset can be used for both county-level and nation-level case count prediction. For this work, the counts per county were used. The Primary Source contained records from 2005 to 2010, and the last five years were treated as a new blocks of records for the creation event experiments. This is an example of a dataset whose variables record the same discrete magnitude (i.e., counts). Although it was not one of the most abundant datasets in terms of records, there was enough information to appreciate a yearly stationary component and relatively low levels of noise (Supplementary Figure 7).

*DS 03* –Global land temperature dataset: The original source of this dataset contained monthly measurements of global land temperatures by country, reported between 1743 and 2013. The measurements were aggregated by the average and variability of temperature measurements within each country each month. Due to a high rate of missing values in previous years, the data used for our experiments started in 1900. The Primary Source dataset included data from 1900 to 1999, and the measurements from this century constituted the block of new records. This dataset was particularly interesting for the creation experiments, as it presented a trend indicating the global temperature increment. Comparing this dataset to DS 04 was of special interest, as the former has the largest number of variables. In contrast, the latter is the dataset with the lowest number of variables and number of records.

*DS 04* –Sales prediction: This dataset provides a company’s revenue, sales, and costs measured monthly from 2015 until March 2020. It is a fairly small and thin dataset compared to the other datasets, and it was included to test the performance of the ML models when the available records are scarce, which makes it a particularly interesting case study for deletion experiments. Moreover, as its variables presented a trend component, it was also interesting for the creation experiments, where new future records are added to a reference dataset with previous records. For the experiments involving the creation of new records, the data from 2015 until 2018 was used as the Primary Source, and the records from 2019 until March 2020 were treated as the Revision set.

*DS 05* –Air Quality dataset: This dataset includes responses from a gas multi-sensor device deployed in an Italian city from 2004 to April 2005. Hourly response averages and gas concentration references from a certified analyser were recorded. For the experiments involving the creation of new records, the data from 2004 was used as the Primary Source, and measurements from 2005 were treated as the new records. These dataset variables do not present any increasing or descending trend but show a daily seasonal component and higher noise levels than the other datasets.

*DS 06* –Ozone Level Detection dataset: This dataset includes ground ozone level data collected from 1998 to 2004 in the Houston, Galveston, and Brazoria areas. The dataset focused on eight-hour peaks of ozone levels above a certain threshold. This work used data from 1998 to 2001 as the Primary Source. Its variables present a yearly seasonal component, and it also displays higher levels of variability.

*DS 07* –Dublin footfall counts dataset: This dataset contains pedestrian footfall counts recorded in Dublin, Ireland, for 30 streets during 2022. Three variables were recorded for each street: the number of people passing in, the number of people going out, and the total number of people passing by the street regardless of their direction. It is an example of a dataset without a specific research context to determine the number of time steps for forecasting or the subset of records for the Primary Source. In this case, the first 75% of the records representing the oldest data were used to fit the Primary Source model. This dataset, as DS 02, measured discrete variables (counts of footfalls) instead of continuous ones. Moreover, it also presents an ascending trend in various streets, making it of particular interest for the creation experiments since some data drift could be expected as newer records were compared to the previous reference values.

## Results

**Fig. 2:**
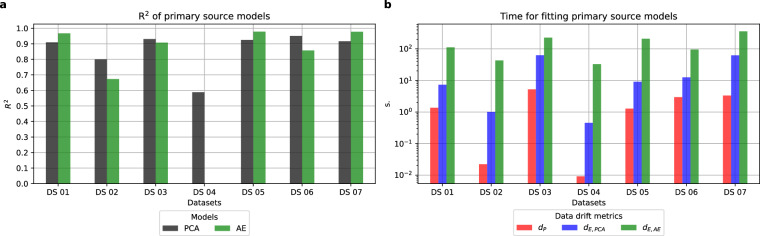
Values of the *R*^2^ coefficients of each ML model (**a**) and of the computation time required to estimate all the elements required for each of the data drift options (**b**) for each of the PS datasets (x-axis).

Before computing the data drift metrics explained in Section 2.2.2, the first step was to select the subsets used as Primary Sources for each dataset and obtain their PCA and AE models. Figure 2 shows the goodness-of-fit coefficients for each model (Fig. 2a) and the time required to fit the models employed by each data drift metric (Fig. 2b) using the PS dataset partitions. Further information about the splines fitting the model between the MSE and the percentages of permuted values can be found in Supplementary Figures 1 and 2.

**Fig. 3:**
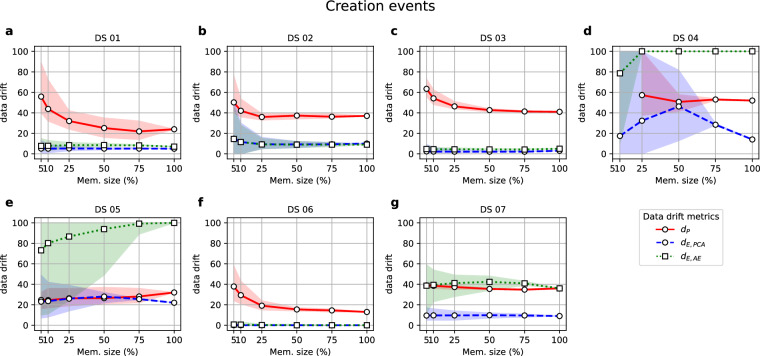
Values of the metrics (y-axis) when new batches of different sizes (x-axis) were added to the Primary Source dataset.

**Fig. 4:**
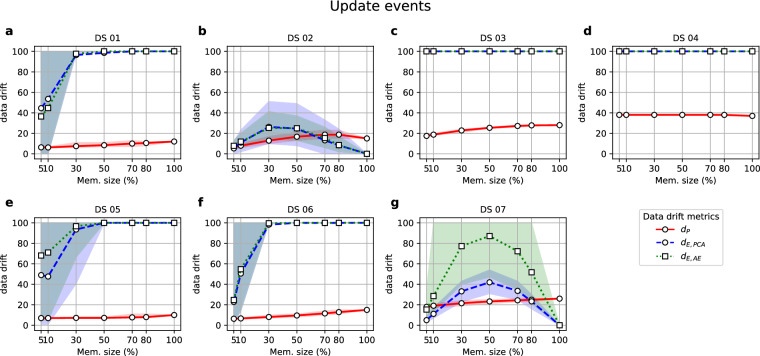
Values of the metrics (y-axis) when they were computed on Revisions with different percentages of variables (x-axis) transformed to a different scale with a cubic root transformation.

Figures 3 to 4 illustrate the relationship between the data above drift metrics and different levels of changes simulated for the datasets. In the following plots, solid lines represent average values over the repetitions of each artefact level. The shaded areas cover the 5th and 95th percentile over the repetitions performed at each level of the simulated scenario, assessing the variability of the values. The times required to compute each data drift metric were also measured and are represented in Supplementary Figures 3 to 5.

Figure 3 displays each dataset’s data drift metric values when creation events are simulated. The experiments employed an initial partition of each dataset as a reference. Subsequently, they updated it by incorporating batches of varying sizes from the remaining dataset partition, which was not utilised in constructing the Primary Source model.

Figure 4 shows the results of update events simulated as the transformation of variables’ scales, as the transformation of values can result in significant shifts that may disrupt the multivariate patterns within the dataset.

**Fig. 5:**
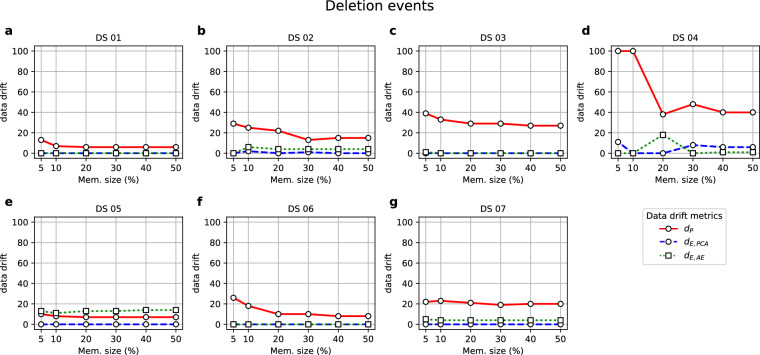
Values of the metrics (y-axis) when they were computed on Revisions obtained by down-sampling the original time series, reducing the sample size (x-axis) of the resulting new versions.

Finally, Fig. 5 demonstrates the impact of deletion events on the data drift metrics.

## Discussion

To interpret the results shown in Figs. 3 to 5, it is important to recall the meaning of each data drift metric. Values of 0 indicate a total agreement between Primary Sources (***X***^(*PS*)^) and Revisions (***X***^(*R*)^) for all data drift metrics. Values of 100 would indicate either a total mismatch of the covariance structure according to *d*_*P*_, or that ***X***^(*R*)^ are as similar to ***X***^(*PS*)^ as completely random values, according to both *d*_*E,PCA*_ and *d*_*E,AE*_ metrics. Values of *d*_*P*_ between 0 and 100 can be interpreted similarly to the absolute value of the correlation coefficient. Non-null values of *d*_*E,PCA*_ and *d*_*E,AE*_ below 50 would indicate that ***X***^(*R*)^ present some changes compared to ***X***^(*PS*)^, but still, most of their values hold information shared by both versions. On the contrary, values of *d*_*E,PCA*_ and *d*_*E,AE*_ above 50 would indicate that more than half of the values within ***X***^(*R*)^ do not present a structure like those seen in ***X***^(*PS*)^.

Overall, the results show the characteristics of the different approaches to quantify the data drift in the context of various events and datasets. First, there is a model-building stage when the Primary Source data is used to fit the ML models. The outcomes of this stage are shown in Fig. 2, proving that both ML models used for the proposed data drift metrics fitted the datasets acceptably (Fig. 2a), with the noticeable exception of DS 04, which shows a decay in the goodness-of-fit for both the PCA and the AE, especially for the latter. This result already highlights the need to assess the goodness of models for each specific dataset, making the necessary adjustments if required. This misfitting for DS 04 must also be considered when evaluating the upcoming results, illustrating the performance of the data-drift metrics, as the results obtained, particularly by the AE model, would invalidate any further results based on such a model.

On a related note, in terms of the time required by each data drift metric, there are differences between the model-building phase shown in Fig. 2b and the execution time obtained for the new version of the datasets in the creation, update, and deletion experiments. When the models are fitted using the Primary Source dataset partition, the *d*_*P*_ requires less than a second in most cases, followed by the *d*_*E,PCA*_ approach, which generally is one order of magnitude faster than the *d*_*E,AE*_ metric. Yet, the computation time for newer versions (Supplementary Figures 3 to 5) is generally shorter and never reaches the order of seconds. In these cases, *d*_*E,PCA*_ is the fastest approach, followed by *d*_*PCA*_ and *d*_*E,AE*_, which alternate the slowest marks depending on the dataset and the events being simulated. The following sections will address the results of each simulated event in more detail.

### Creation events

Results from the creation events aimed to answer two questions: *(i) Would data drift metrics yield values between 0 and 50, indicating a major agreement between revisions and primary sources, even if some disparate batches are present in the revision?*; and *(ii) Would non-null values of data drift metrics be aligned with actual changes appreciated in seasonal or trend components of time series?*

Results from Fig. 3 indicate that the *d*_*P*_ metric generally exhibits higher values than *d*_*E,PCA*_ and *d*_*E,AE*_, and between the latter two, *d*_*E,PCA*_ is the one being closer to yield data drift values below 50. Yet, *d*_*E,PCA*_ values are far from null in most cases, which would be interpreted as the Revision datasets being somewhat different to the Primary Source but still alike for most of the values. This leads to the second research question, seeking the interpretability of these results.

**Fig. 6:**
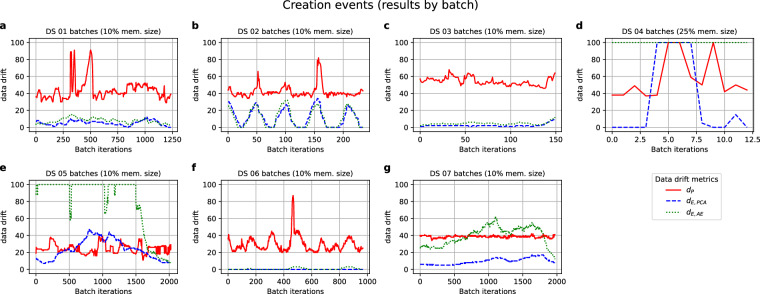
Values of the metrics (y-axis) when computed on batches of a 10% of the Revision subset for each one of the datasets, except for DS 04, with batches of a 25% size.

To interpret data drift values from Fig. 3, we inspected the outcomes at low levels of memory size, i.e., we increased the time resolution to check if the data drift values showed some meaningful evolution with time. One aspect worth mentioning is the higher variability of data drift values for smaller batch sizes across all datasets, particularly noticeable for DS 02 and DS 05 (Fig. 3b and e, respectively). On the one hand, this evident impact of batch sizes on the metrics’ variability is due to a decrease in the available time windows used for different repetitions as the batch sample size increases. For instance, the metrics’ values for a 100% batch memory size are the outcomes over all the records in the Revision dataset. Nevertheless, the variability can also be increased due to the presence of disparate batches. Figure 6 illustrates the data drift values obtained for each one of the batches at a high time resolution (10% of memory size for all datasets except for DS 04, with batch sizes of 25% of the memory size as 5% and 10% of memory size did not reach samples with more than a single observation).

When examining the batch-wise data drift values in Fig. 6, it becomes apparent that both *d*_*E,PCA*_ and *d*_*E,AE*_ reflect patterns observed in the time series and extracted in either the trend or the seasonal components during the exploratory analysis (see Supplementary Figures 6 to 12). The following paragraphs will address some of the most insightful ones.

DS 02 (Fig. 6b) shows five peaks in the count of Hungarian Chickenpox cases, lasting around 50–60 iterations each, aligned with the yearly seasonal component (see Supplementary Figure 7). These results suggest that the appearance of drifted batches corresponds to the peaks in counts. Still, results for *d*_*E,PCA*_ and *d*_*E,AE*_ converge to lower values with bigger batches (Fig. 3b), as these dynamics with peaks are part of the normal patterns already present in the Primary Source data.

DS 04 combines two aspects; first of all, a scarcity of data that has already affected PS models by yielding the lowest goodness-of-fit of all (Fig. 2a), which is null for the Autoencoder and explains why new batches are constantly seen as random noise (i.e., *d*_*E,AE*_ = 100). The second aspect is an ascending trend in all the DS 04 features that results in values within the Revision set out of the range seen for the Primary Source set (see Supplementary Figure 9). This factor explains why some new batches trigger major updates (i.e., *d*_*E,PCA*_ = 100 as well for low memory size levels), as both the data substantially varies and the PS model did not have enough information to capture the dynamic of the dataset (explaining also the high *d*_*E,AE*_ values). Therefore, when new values out of range come, even if they respect the general patterns and dynamics of the data, they will yield high data drift values.

**Fig. 7:**
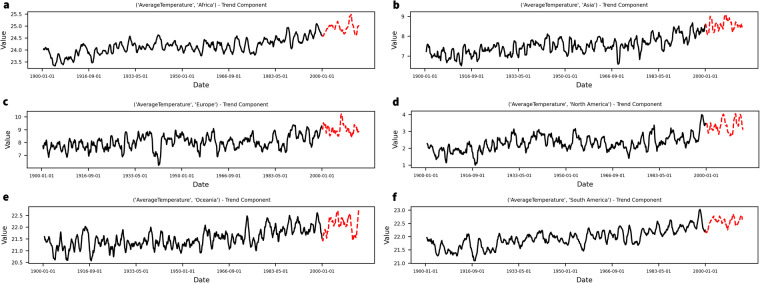
Examples of values of the trend component for variables referring to continents from the DS 03 used for the PS (black) and the Revision batches (red).

It is of special interest to juxtapose the case of DS 04 with DS 03 and DS 07. These two latter datasets also show clear ascending trends in many of their variables, but they are much richer in data. When enough data is available to fit the PS models, the result is quite different. As can be seen in Fig. 6c, both *d*_*E,PA*_ and *d*_*E,AE*_ values show a slight increase towards the last batches, which is aligned with extreme values derived from the globally ascending trend of temperatures worldwide (Fig. 7). Nonetheless, the availability of enough data makes both the AE and PCA models more robust to such changes, yielding increased data drift values but still recognising the patterns within the data.

**Fig. 8:**
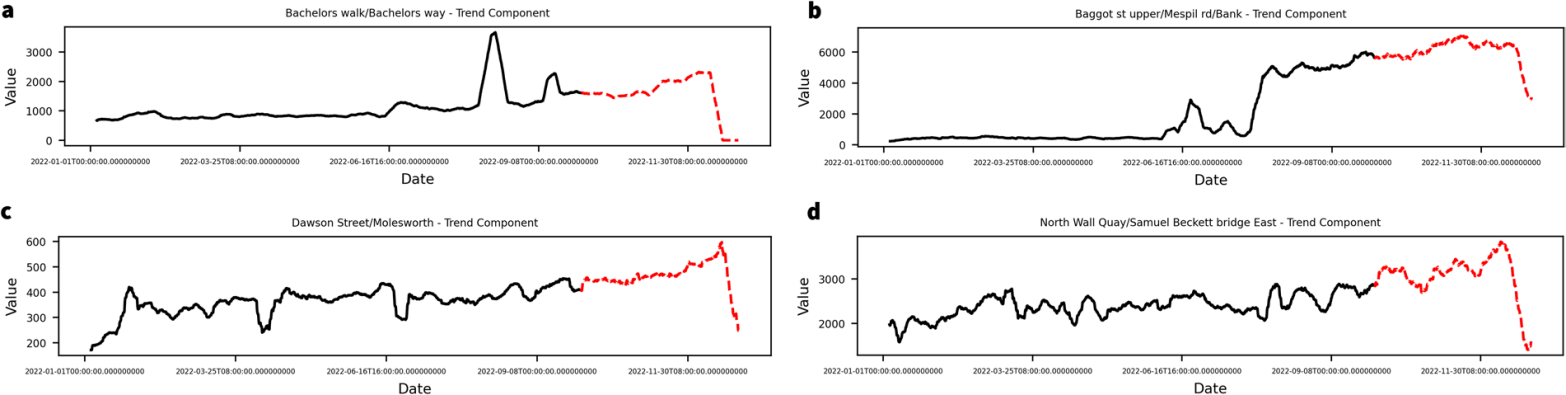
Four examples of increasing trend components for variables from the DS 07 used for the PS (black) and the Revision batches (red).

Yet, the case of DS 07 showcases that *d*_*E,PCA*_ is more robust to the appearance of extreme values than *d*_*E,AE*_, which triggers major updates for this dataset as well. Figure 8 shows some of the streets within DS 07 showing ascending trends ended by a dramatic decay in footfall counts by the end of 2022. The ubiquity of this decay, which is also appreciated in the decay of *d*_*E,AE*_ in Fig. 6g, might indicate the need for a sensor maintenance check. Still, most streets of the Revision batches showed footfall count values out of the PS range but aligned with an ascending trend already present in the PS set, and this dynamic was only successfully captured by *d*_*E,PCA*_.

DS 05 also showed more variability in Fig. 3e than the rest of the datasets. Supplementary Figure 10 shows that the trend component for some variables in DS 05 (namely AH, NO2(GT), PT08.S1(CO) and T) exhibits a peak that resembles, to some extent, the dynamic appreciated in Fig. 6e for *d*_*E,PCA*_. Nonetheless, the reasons for *d*_*E,PCA*_ and *d*_*E,AE*_ trajectories in Fig. 6e are less clear, suggesting that batches might be disparate in a multivariate manner rather than in a univariate one, which would not be visible in plain sight.

Finally, cases such as DS 01 and DS 06, where no extreme values were present in the Revision set, at least in plain sight, show stable and low values (far below 50) for both *d*_*E,PCA*_ and *d*_*E,AE*_, whereas this is not the case for *d*_*P*_, which shows abrupt changes along the incoming batches. Whereas these changes in *d*_*P*_ for DS 06 (Fig. 6f) seem to relate to the seasonal component of variables (see Supplementary Figures 11 and 12), it is harder to interpret its changes for DS 01 (Fig. 6a).

In conclusion, as per the second question formulated at the beginning of this section, *d*_*E,PCA*_ seems to yield more interpretable data drift values than *d*_*P*_ and *d*_*E,AE*_. Another aspect to mention is that despite the qualitative agreement between the dynamics of *d*_*E,PCA*_ and *d*_*E,AE*_ appreciated in Figs. 3 and 6, the latter metric shows higher values of average data drift, especially for DS 05 and DS 07 (Fig. 3e and g, respectively). One possible explanation could be that the AE tend to overfit more than PCA. Noisier datasets, such as DS 05 and DS 07, could therefore yield a higher data drift due to the noisier nature of these signals. Consequently, one potential solution may involve augmenting the noise parameter employed during AE fitting when dealing with noisy datasets. By doing so, the model might exhibit more robustness to such variations, leading to lower data drift values.

### Update events

Next, the update-event experiments showcase the outcomes when the dataset values are altered. Results from the update events aimed to answer the following two questions: *(i) Would data drift metrics reach values of 100, indicating that a major update of the version number is needed, revealing a change of units in the measurements?*; and *(ii) Would data drift metrics show consistent outcomes to shifts in scale regardless of the variables’ magnitudes?*

The results in Fig. 4 show that the *d*_*P*_ metric generally demonstrates lower values than *d*_*E,PCA*_ and *d*_*E,AE*_ metrics. This discrepancy can be attributed to the fact that the scale of the dataset values directly influences the latter two metrics. Conversely, a change in ranking does not necessarily imply a breakdown of the correlation structure, as correlations operate on normalised variable values. As long as the underlying patterns remain intact, the yielded ***P***^(*R*)^ loading matrix will be similar to the ***P***^(*PS*)^ loading matrix, thereby explaining the lower values of the *d*_*P*_ metric.

Thereby, as per the first question formulated at the start of this section, the results from Fig. 4 would favour the use of *d*_*E,PCA*_ or *d*_*E,AE*_ metrics. Both show values of 100, or at least above 50, when over 30% of variables have been shifted. As per the second question, they seem to be heavily affected by the nature of the variables, showing a completely different performance for DS 02 and DS 07 (Fig. 4b and g, respectively), which have discrete variables instead of continuous ones. This suggests the need for special modifications for the data drift metrics to deal with different types of variables. Still, this could be easily accommodated by the framework described in Section 2.1.

### Deletion events

Finally, simulations of deletion events aimed to answer the following question: *Would data drift metrics yield null values, indicating that previously seen records are detected as so?* Fig. 5 illustrates that the *d*_*P*_ metric shows a higher sensitivity to information loss than the other two metrics, *d*_*E,PCA*_ and *d*_*E,AE*_. This is expected since the model fitted with the down-sampled Revision set may be significantly affected by the information loss, leading to a higher data drift value. This effect is exacerbated for DS 04 (Fig. 5d), which has the smallest sample size, and therefore is more affected by the information loss. Besides, even for datasets DS 01 (Fig. 5a), DS 05 (Fig. 5e) and DS 07 (Fig. 5g), where *d*_*P*_ does not seem to vary according to the memory size, it yields values consistently higher than *d*_*E,PCA*_.

On the contrary, both *d*_*E,PCA*_ and *d*_*E,AE*_ metrics do not require retraining the model and instead project the records remaining in the Revision dataset onto the Primary Source models, explaining the lower data drift values for both metrics over all the percentages of deleted records and datasets. Among these two metrics, *d*_*E,PCA*_ exhibits more stable results, accurately recognising the records as part of the reference dataset used as the Primary Source for all datasets except for DS 04, which shows an increase from 30% of deleted records. This suggests that the underlying PCA model employed by *d*_*E,PCA*_ is more robust to information loss than the AE-based approach used by *d*_*E,AE*_.

In short, answering the research question posed above, the results from Fig. 5 show that the *d*_*E,PCA*_ metric yields the lowest values across all datasets and downsampling percentages. Interestingly, it is worth noting that *d*_*E,AE*_ shows higher data drift values for DS 05 and DS 07, as observed in the creation event experiments (Fig. 3e and g, respectively). As mentioned earlier, this could be attributed to the noisy nature of these datasets, which affects the AE performance. Increasing the AE robustness by exposing it to training datasets with higher noise factors during the model-building stage could mitigate this issue and lead to lower data drift values.

### Concluding remarks

In conclusion, in this study, we proposed a standardised data versioning framework that addresses the limitations of existing approaches, particularly in terms of findability, reusability, and recognition of data versions. By incorporating principles from the RDA and the DataCite Metadata Schema, we aimed to enhance the tracking of changes in data over time, facilitate reproducibility, and foster collaboration. One of the key limitations of current data versioning frameworks is the reliance on citations from journal papers to make new data versions discoverable. Users often search for datasets based on specific attributes or features rather than following paper citations. Moreover, the lack of tools to understand the differences between data versions results in inefficient discovery and reuse, leading to sub-optimal resource utilisation. We have proposed a standardised data versioning nomenclature to overcome these limitations based on the widely recognised software versioning format of “major.minor.patch”. This terminology aligns with the foundational principles of effective data versioning practices and complies with the existing fields in the DataCite schema.

Within this framework, we have introduced the concept of a data drift metric as the “minor” term of the version tag. Data drift refers to changes or deviations in the statistical properties or distributions of data over time. By quantifying data drift and including it as a standard metric within the DataCite metadata schema, researchers and data consumers can gain insights into the extent of modifications within datasets, making informed decisions regarding data usability. We have explored and evaluated three data drift metrics, namely *d*_*P*_, *d*_*E,PCA*_, and *d*_*E,AE*_. These metrics leverage ML models to quantify data drift and capture dataset changes.

Our experiments have revealed important insights into the performance of these metrics under different scenarios, including creation, update, and deletion events. According to the performed experiments, the *d*_*E,PCA*_ metric showed bounded values below 50 in creation event experiments, showing interpretable values that consistently increased along with time series changes (Figs. 3 and 6). As discussed in Section 4.1, at a higher time resolution (i.e., for small batch sizes), the metric successfully reflected variations, if present. Still, overall, the PS PCA model seemed to successfully capture the general patterns of the data, keeping *d*_*E,PCA*_ values below 50 provided that Revision sets were batches long enough in time, representing complete seasonal patterns, and not small batches with only the extreme values within a cycle. Next, Section 4.2 discussed the update experiments performing scale transformations (Fig. 4), concluding that the *d*_*E,PCA*_ consistently triggered major updates of the data version (i.e., *d*_*E,PCA*_ = 100) when more than 30% of the variables had suffered a scale shift. Nevertheless, this outcome was not consistent for datasets with variables with a nature other than continuous (DS 02 and DS 07). Deletion experiments (Fig. 5) also showed that the *d*_*E,PCA*_ metric was the most robust against the loss of information, keeping data drift values equal to 0 for almost all cases, except for DS 04, which showed higher data drift values for Revisions retaining more than 30% of records from the Primary Source set. Nonetheless, the DS 04 was already a special case study showcasing the outcomes when information scarcity compromises the model-building step with the PS. Therefore, results unlike the ones expected for the rest of the datasets could be expected. Finally, regarding computational time, the *d*_*E,PCA*_ approach presents the best trade-off solution, with a model-building time generally under two minutes (Fig. 2b) and execution times (Supplementary Figures 3 to 5) consistently under a second.

For all these reasons, the *d*_*E,PCA*_ metric shows the best trade-off regarding performance interpretability, stability, robustness against information loss, and computation time. Nevertheless, future work is needed to address some of the limitations uncovered by the experiments. First, the *d*_*E,PCA*_ metric presented some inconsistencies when working with non-continuous data sets, as seen with DS 02 and DS 07 in the update experiments performing a cubic root transformation. Some adaptations of PCA to deal with non-continuous datasets could be adopted for such cases. Small datasets with scarce information, such as DS 04, can present some issues, preventing the obtention of a model that is accurate enough. One potential solution could be using data augmentation strategies for such cases during the model-building phase with the Primary Source.

The final suggestion of terms for the standard version tag and their corresponding fields in the DataCite Metadata Schema and their conceptual meaning are summarised in Table 2. Despite this being the final suggestion derived from this work, it has also been shown that the choice of data drift metric plays a crucial role in capturing and understanding data drift phenomena, and researchers should consider the specific characteristics of their datasets and choose the appropriate metric accordingly. Nevertheless, the proposed framework enables the choice of different models as best practices for each dataset, as long as the same technique is applied across all the version history.

**Table 2:** Summary of the final suggestion for the standard terms conforming to the data versioning tags.

Version tag term	Meaning	DataCite field
Patch	Small, specific fixes or corrections applied to the dataset represented by timestamps	“datacite.VersionDate”
Minor	Standardised data drift metric indicating the shift between a data version and a reference point, typically the oldest version sharing the same data model (major term). The metric can be interpreted as the equivalent level of random perturbation seen on the reference dataset	“dataDriftMetric” (custom field created using the “relatedIdentifier” element)
Major	Significant, and substantial changes to the dataset, often involving substantial modifications, additions, and deletions to the data structure, schema, or underlying data model	“datacite.SchemaVersion”, “datacite.DataModelDescription” and “datacite.DataModelIdentifier”

Overall, our proposed standardised data versioning framework, coupled with incorporating data drift metrics, offers a comprehensive solution to improve the findability, reusability, and recognition of data versions. By leveraging ML techniques to quantify data drift and adopting the “major.minor.patch” format, researchers can effectively track changes in datasets, facilitate comparisons between versions, and make informed decisions regarding data usability and integration into workflows and analyses. While simpler tools for data comparison exist, these proposals using ML models are a first step that paves the way for more sophisticated tools that interact with such models and obtain further information. This is evident in the downsampling (Deletion event) experiments, which provide evidence about the resilience of data-drift metrics based on machine learning models against traditional comparison tools such as “diff” commands. New tools exploiting the ML tools implemented to enable the proposed data versioning standard could be used for further purposes, such as interpreting the differences between datasets or integrating more sophisticated mechanisms for data quality assurance.

The proposed technique could have a diverse user base, catering to key stakeholders in the research data ecosystem. First, data authors (data producers) could benefit from a systematic approach to monitor and quantify data drift, ensuring data quality and reproducibility. Secondly, if such a tool was implemented in a repository (data hosters), its managers would gain valuable insight for assessing dataset evolution within repositories, enabling effective curation decisions. Thirdly, researchers and data curators (data consumers) would find utility in understanding dataset changes, facilitating comparisons, and ensuring reliable datasets for analysis. Collectively, our technique could contribute to enhanced data management practices, supporting collaborative research endeavours and augmenting the overall reliability and usability of research data. For this reason, we consider that the optimal step would be its implementation as a service in data repositories, where it can serve all the aforementioned roles and user profiles engaged in data-intensive projects.

Further work may include generalising the proposed framework to datasets, including categorical variables, by expanding the ML models to adaptations that deal with such data. For instance, Generalised Simultaneous Component Analysis (GSCA)^27^ can be applied as a more generalistic PCA framework for datasets with variables of mixed nature. Extending the framework to lower levels of granularity, i.e., to each independent record, is another clear path for future work, enabling the versioning of streaming datasets with single-record creation events. On this matter, the PCA framework already presents established schemes from Statistical Process Control for the online detection of unsupervised anomalies^18,28^. Finally, future research should also explore alternative data drift metrics, especially for datasets of reduced dimensions, given the results obtained with dataset DS 04 (Figs. 2, 3, 6, and 5). The proposed system’s applicability to data models other than time series and potential extensions towards Continual Learning frameworks assessing the relationship of the proposed data drift metrics with changes in the ML models based on such datasets should also be investigated.

### Supplementary information

Supplementary Material

## Data Availability

All experiments have been modelled and programmed using Python and open datasets. In the interest of full reproducibility, the code and datasets to reproduce the experiments are included in a .zip file available at Figshare^29^ and in the public GitHub repository^30^. The results have been obtained using Python 3.10.9 and the packages used and their corresponding versions are: keras 2.11.0, keras_tuner 1.3.0, matplotlib 3.7.1, numpy 1.23.5, pandas 2.0.3, pytest 7.4.0, scikit_learn 1.2.2, scipy 1.10.1, seaborn 0.12.2, statsmodels 0.13.5, tensorflow 2.11.0, and openpyxl 3.11.1. The instructions to run the scripts for the demos or the experiments are available in the README.md file included both in the .zip file at Figshare^29^ and in the GitHub repository^30^.

## Data availability

The authors declare that the data supporting the findings of this study are available within the paper and its Supplementary Information files^29^. Should raw data files be needed in another format, they are available from the original sources, allocated in the third-party repositories listed below:

• DS 01, SML2010, available from^31^;

• DS 02, Hungarian chickenpox cases, available from^32^;

• DS 03, Global land temperature, available from^33^;

• DS 04, Sales prediction, available from^34^;

• DS 05, Air quality, available from^35^;

• DS 06, Ozone level detection, available from^36^; and

• DS 07, Dublin footfall counts 2022, available from^37^.
