# Supplementary material for: Standardised Versioning of Datasets: a FAIR–compliant Proposal
Source: Sci Data. 2024 Apr 9;11:358. doi: 10.1038/s41597-024-03153-y (PMC11003959; doi:10.1038/s41597-024-03153-y)
Supplement: Supplementary file 1 — Supplementary Material [file 41597_2024_3153_MOESM1_ESM.pdf]

Supplementary Materials for “Standardised Versioning of Datasets: a FAIR–compliant Proposal”

Alba González–Cebrián[1,*], Michael Bradford[1], Adriana E. Chis[1], and Horacio González–Vélez[1]

[1]Cloud Competency Centre, National College of Ireland, Dublin, Dublin, Ireland
[*]corresponding author(s): Alba González–Cebrián (alba.gonzalez-cebrian@ncirl.ie)

Contents

1 Visualisation of the splines fitting **1**
Supplementary Figure1 MSE (x-axis) and corresponding permutation percentage (y-axis) obtained over a hundred repetitions with the PCA models fitted on each one of the PS subsets of the datasets from Table 1. Red dashed lines represent the prediction obtained by the fold of 10 splines for the average MSE values obtained when each level of permutation was simulated on the training dataset. . . . 2
Supplementary Figure2 MSE (x-axis) and corresponding permutation percentage (y-axis) obtained over a hundred repetitions with the AE models fitted on each one of the PS subsets of the datasets from Table 1. Red dashed lines represent the prediction obtained by the fold of 10 splines for the average MSE values obtained when each level of permutation was simulated on the training dataset. . . . 3

2 Execution time of the experiments **4**
Supplementary Figure3 Average (solid line) time required to compute the data drift metrics with each level of batch size addition for the creation event experiments shown in Figure 2. . . . 4
Supplementary Figure4 Average (solid line) time required to compute the data drift metrics with each level of batch size transformation for the update experiments shown in Figure 3. . . . 5
Supplementary Figure5 Average (solid line) time required to compute the data drift metrics with each level of information retained for the deletion experiments shown in Figure 4. . . . 5

3 Exploratory analysis with time series decomposition **6**
Supplementary Figure6 Time series decomposition for variables from dataset DS 01. . . . 7
Supplementary Figure7 Time series decomposition for variables from dataset DS 02. . . . 8
Supplementary Figure8 Time series decomposition for variables from dataset DS 03 referring to continents. . . . 9
Supplementary Figure9 Time series decomposition for variables from dataset DS 04. . . . 9
Supplementary Figure10 Time series decomposition for variables from dataset DS 05. . . . 10
Supplementary Figure11 Time series decomposition for variables from dataset DS 06 (KI to T3). . . . 11
Supplementary Figure12 Time series decomposition for variables from dataset DS 06 (T4 to T18). . . . 12
Supplementary Figure13 Time series decomposition for variables from dataset DS 06 (T19 to V70). . . . 13
Supplementary Figure14 Time series decomposition for variables from dataset DS 06 (V85 to WSR12). . . . 14
Supplementary Figure15 Time series decomposition for variables from dataset DS 06 (from WSR13 to WSR_PK). . . . 15
Supplementary Figure16 Time series decomposition for variables from dataset DS 07 (from Bachelors’ walk to Henry Street). . . . 16
Supplementary Figure17 Time series decomposition for variables from dataset DS 07 (from Mary Street to Westmoreland Street West-Carrolls). . . . 17

1 Visualisation of the splines fitting

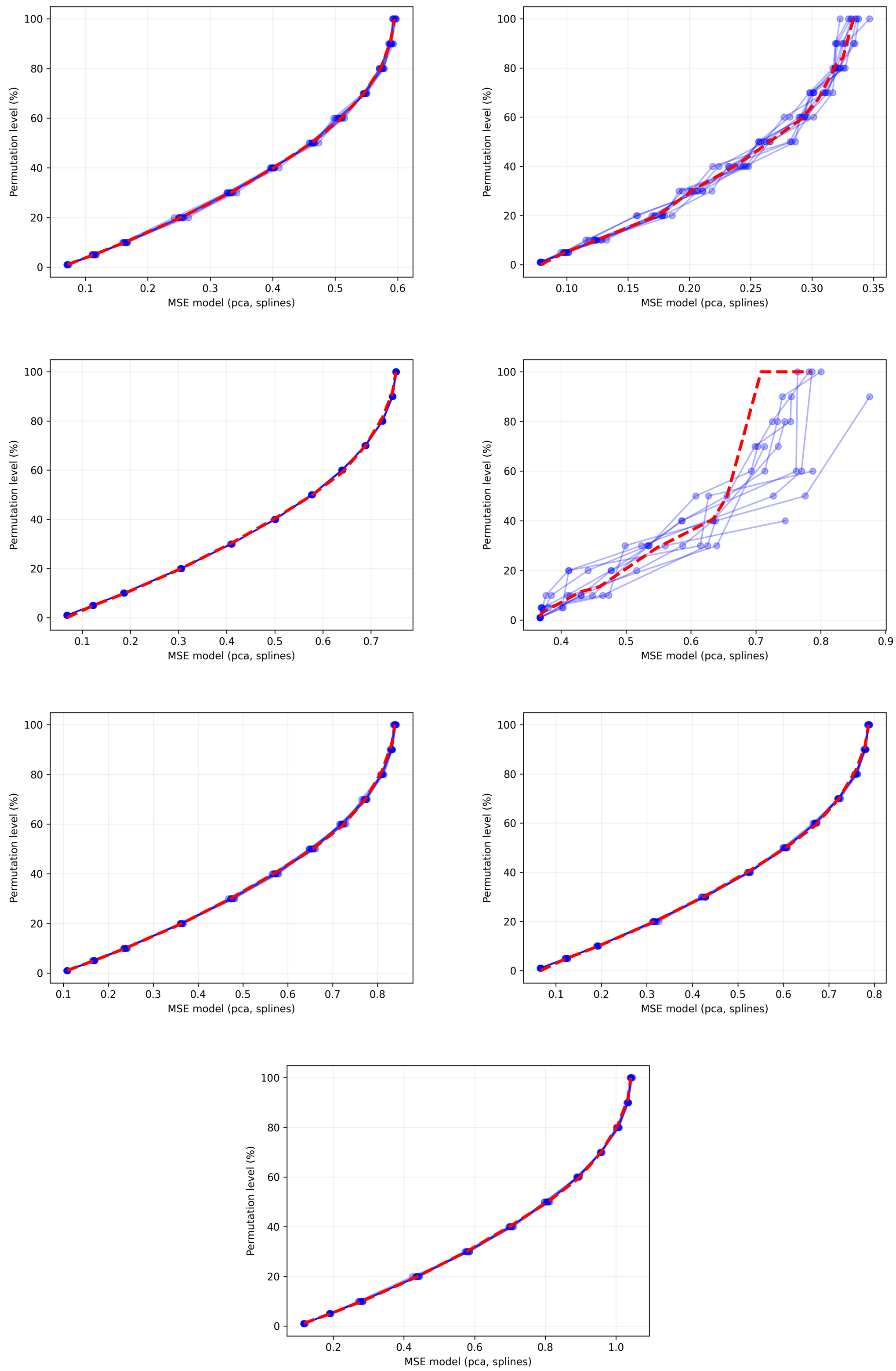

Supplementary Figure 1. MSE (x-axis) and corresponding permutation percentage (y-axis) obtained over a hundred repetitions with the PCA models fitted on each one of the PS subsets of the datasets from Table 1. Red dashed lines represent the prediction obtained by the fold of 10 splines for the average MSE values obtained when each level of permutation was simulated on the training dataset.

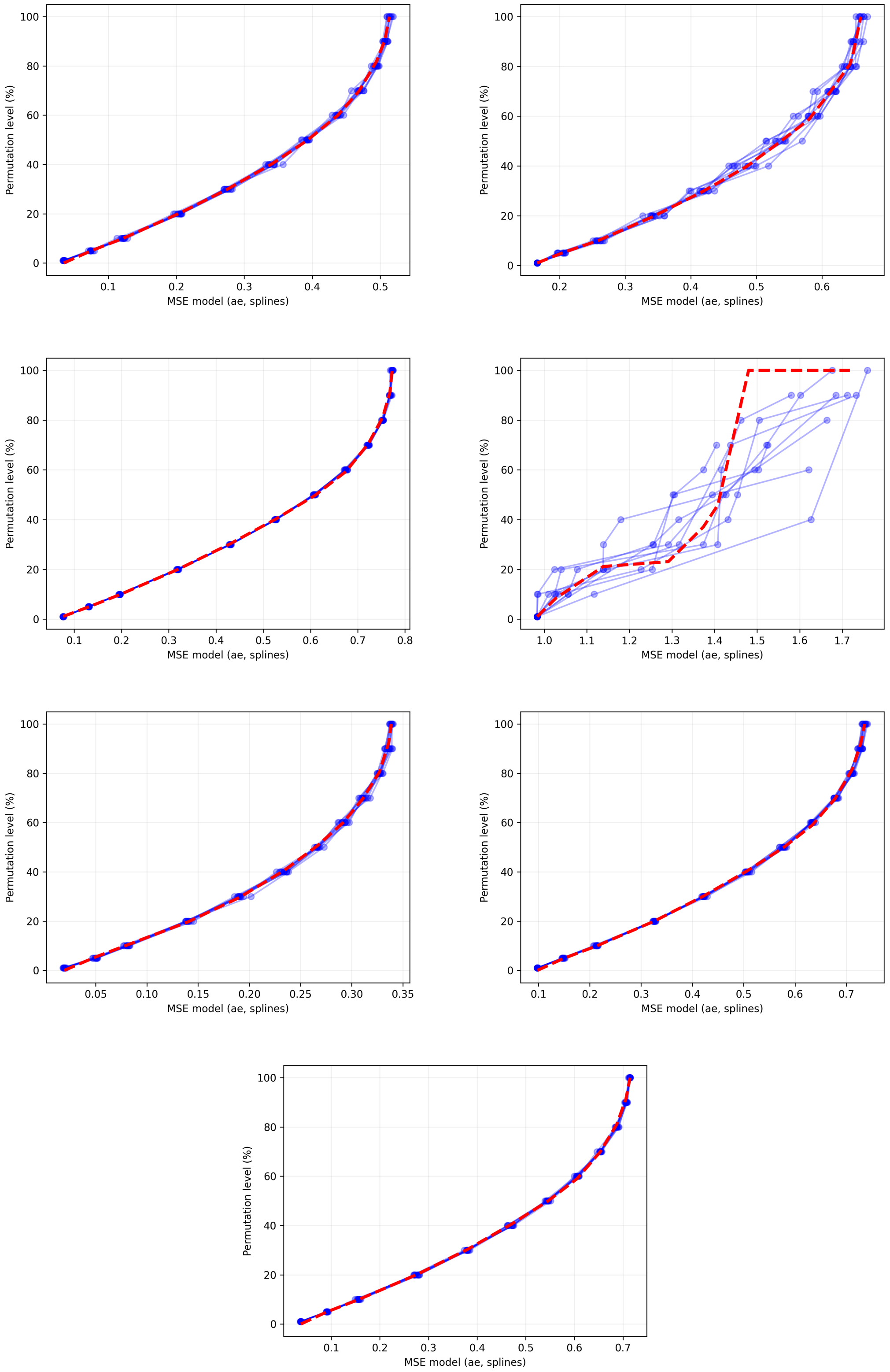

Supplementary Figure 2. MSE (x-axis) and corresponding permutation percentage (y-axis) obtained over a hundred repetitions with the AE models fitted on each one of the PS subsets of the datasets from Table 1. Red dashed lines represent the prediction obtained by the fold of 10 splines for the average MSE values obtained when each level of permutation was simulated on the training dataset.

2 Execution time of the experiments

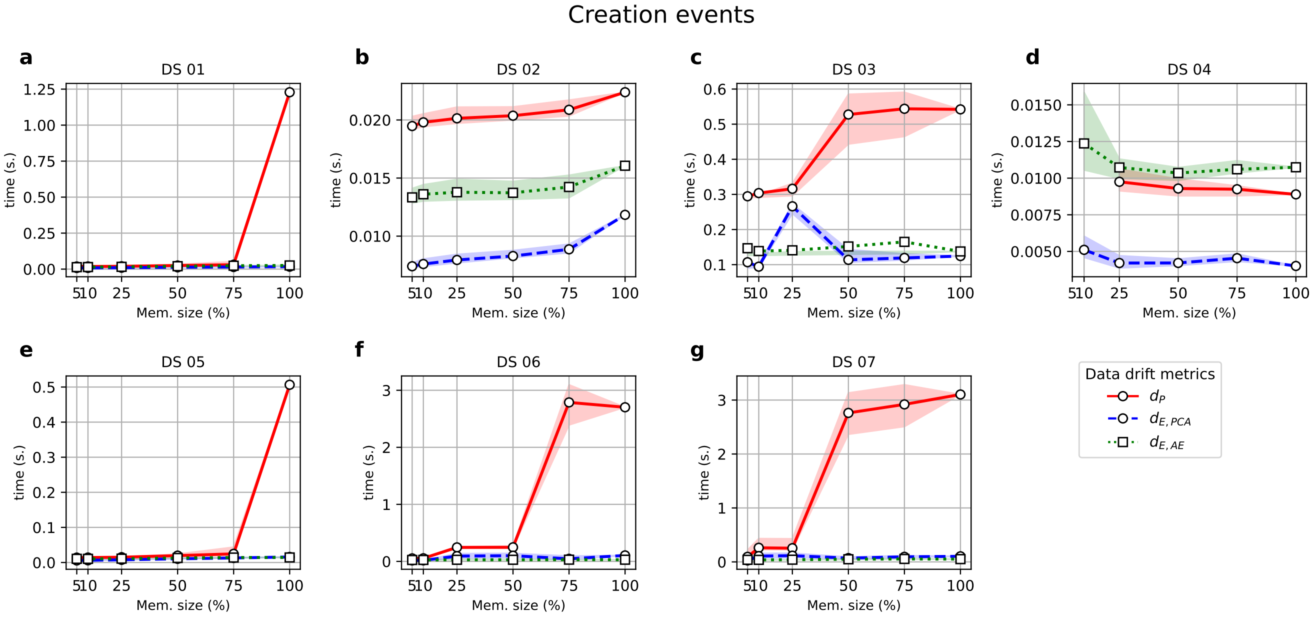

Supplementary Figure 3. Average (solid line) time required to compute the data drift metrics with each level of batch size addition for the creation event experiments shown in Figure 2.

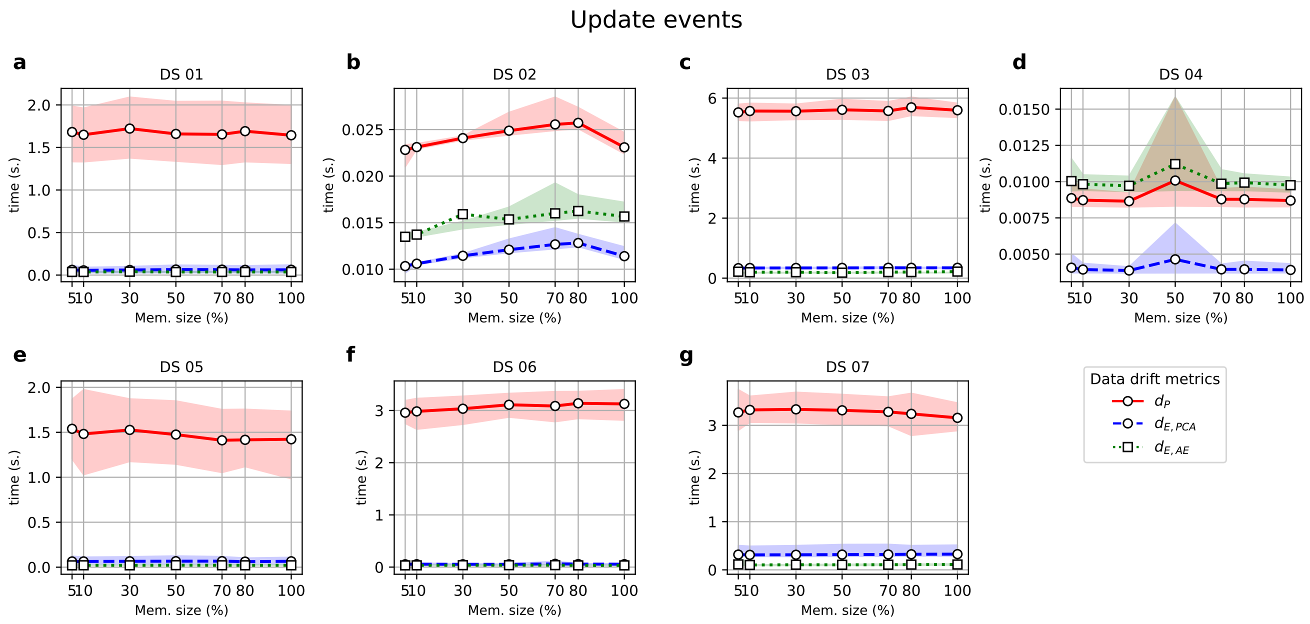

Supplementary Figure 4. Average (solid line) time required to compute the data drift metrics with each level of batch size transformation for the update experiments shown in Figure 3.

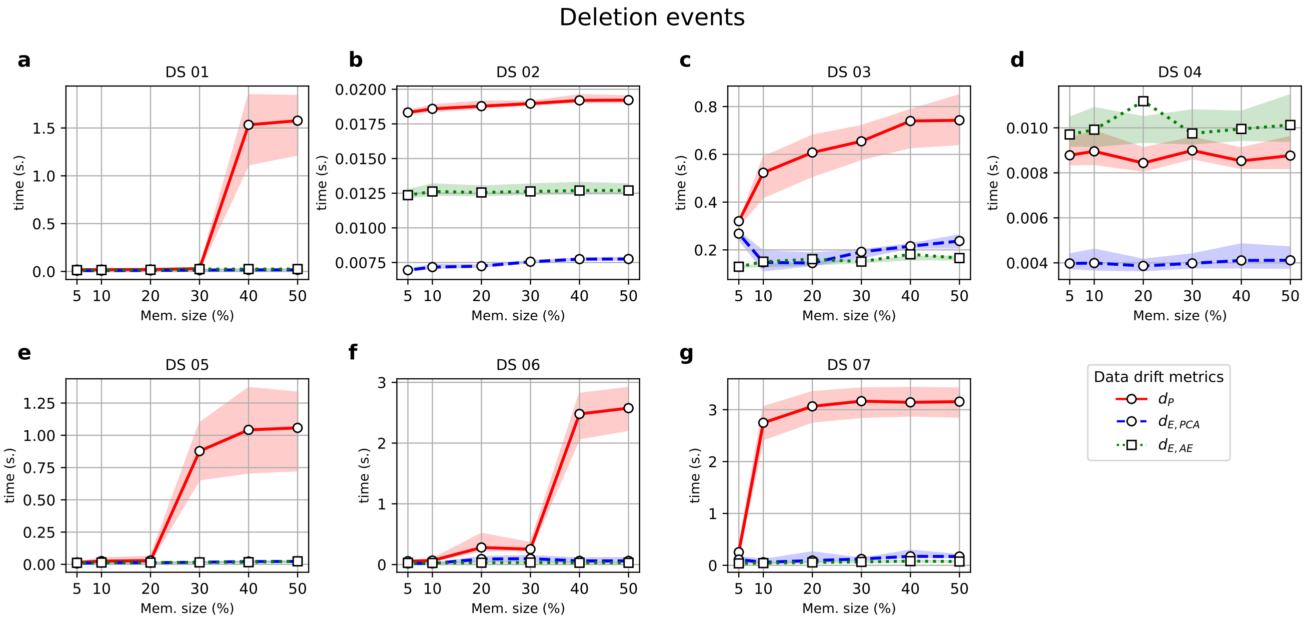

Supplementary Figure 5. Average (solid line) time required to compute the data drift metrics with each level of information retained for the deletion experiments shown in Figure 4.

3 Exploratory analysis with time series decomposition

The time series decomposition separates the original data ($Y(t)$) into a combination of different components:

- Trend ($T(t)$): reflects the long-term progression of the series, for instance, when there is a persistent increasing or decreasing direction in the data, although it does not need to be linear, and it can also contain the cyclic variations;
- Seasonal ($S(t)$): exists when seasonal factors influence a time series over a fixed and known period;
- Cyclic ($C(t)$): reflects fluctuations that depend on the nature of the time series and are not periodic;
- Error ($e(t)$): describes irregular dynamics that the other components left unexplained.

In our case, the decomposition was performed by the Python function *seasonal_decompose* from the *statsmodels.tsa.seasonal* module. It can represent a time series as:

- $Y(t) = T(t) + S(t) + e(t)$ if the additive model is used;
- or as $Y(t) = T(t) \cdot S(t) \cdot e(t)$ if the multiplicative model is used instead.

The results are obtained by first estimating the trend by applying a convolution filter to the data. The trend is then removed from the series, and the average of this de-trended series for each period is the returned seasonal component. As can be seen, it yields a naive decomposition, grouping the trend and cyclic components, and more sophisticated methods should be preferred for better and more accurate time series decomposition. However, these results were merely used to understand better the results obtained by the data drift metrics afterwards, and their purpose was not to perform an in-depth analysis.

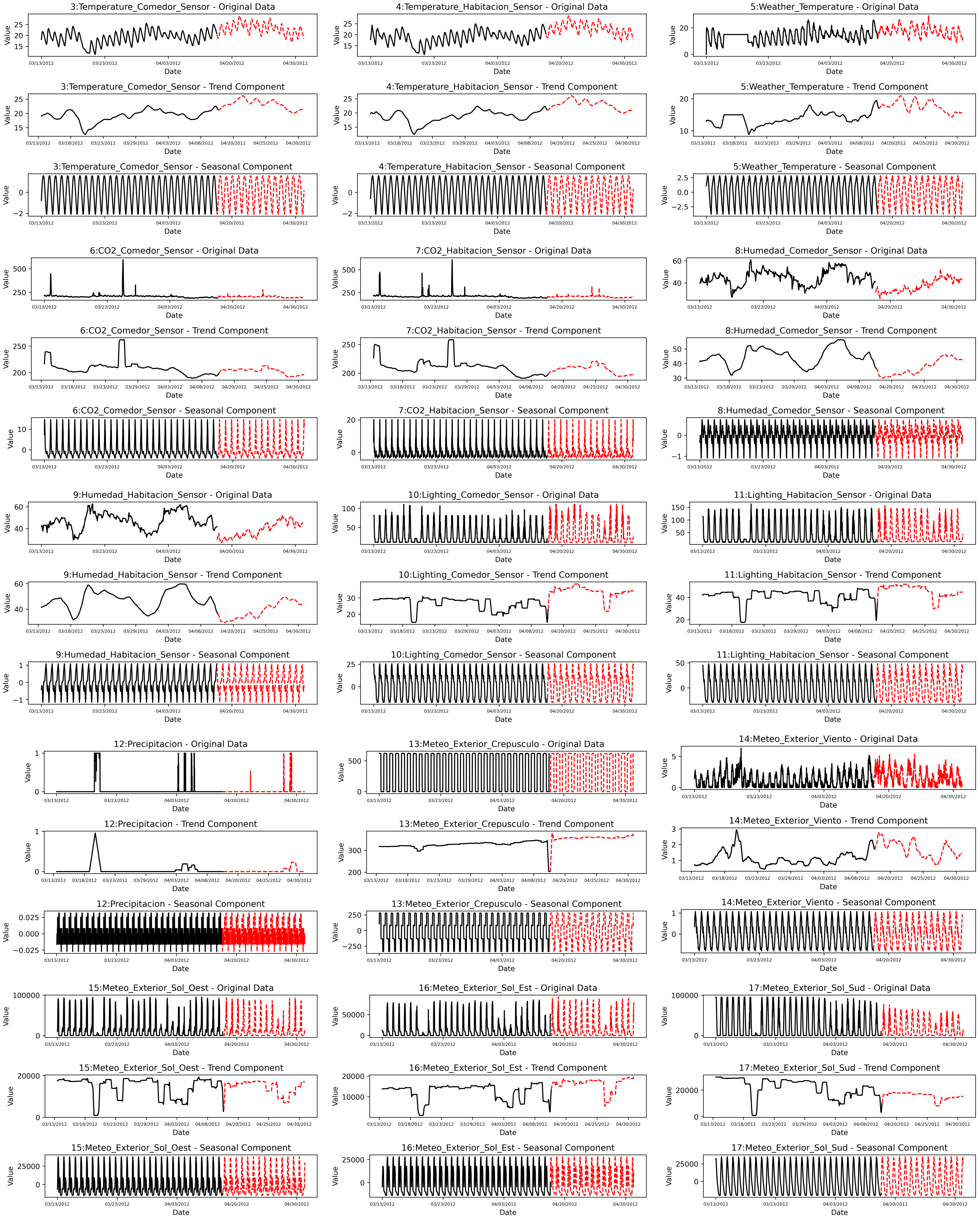

Supplementary Figure 6. Time series decomposition for variables from dataset DS 01.

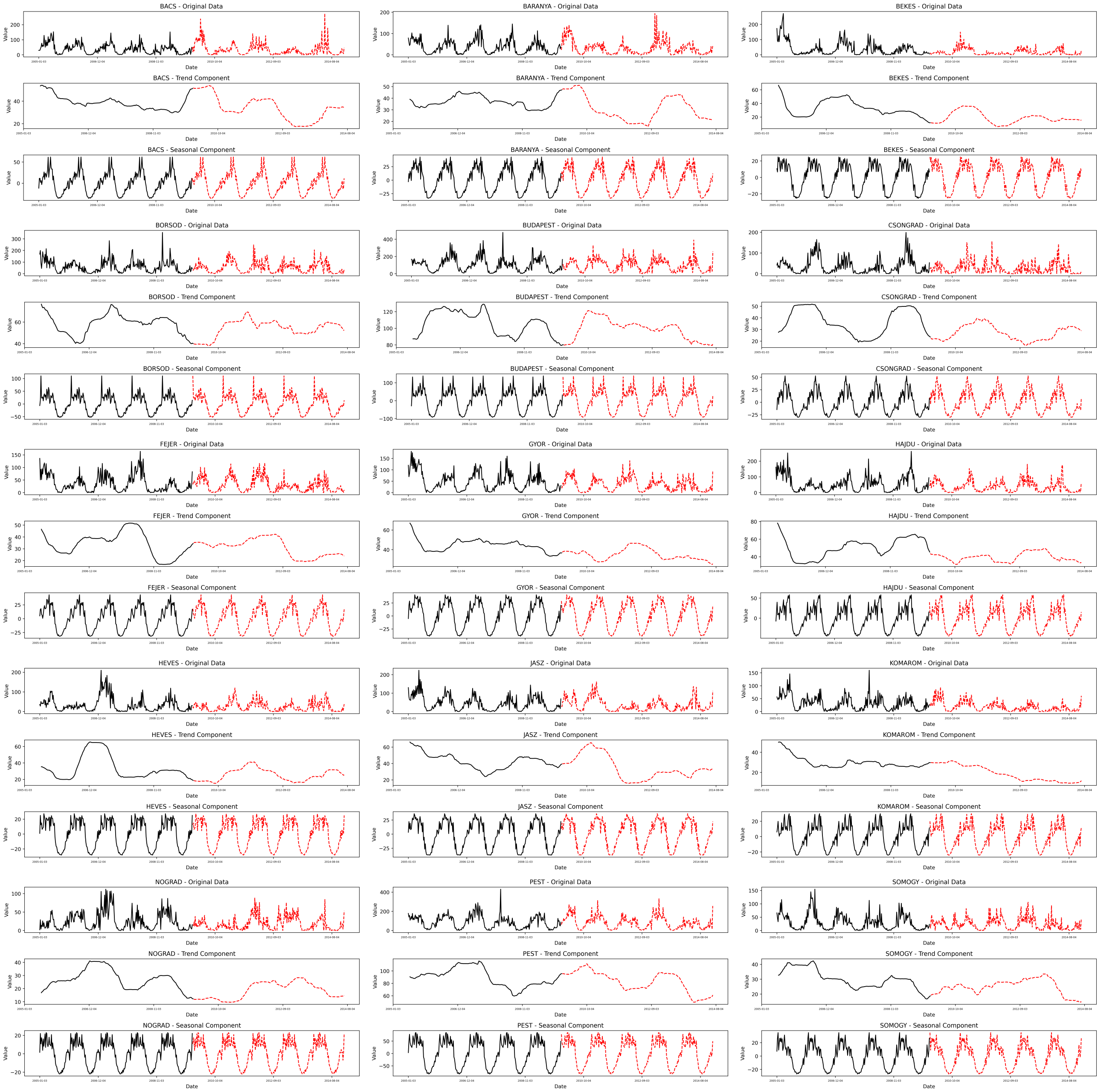

Supplementary Figure 7. Time series decomposition for variables from dataset DS 02.

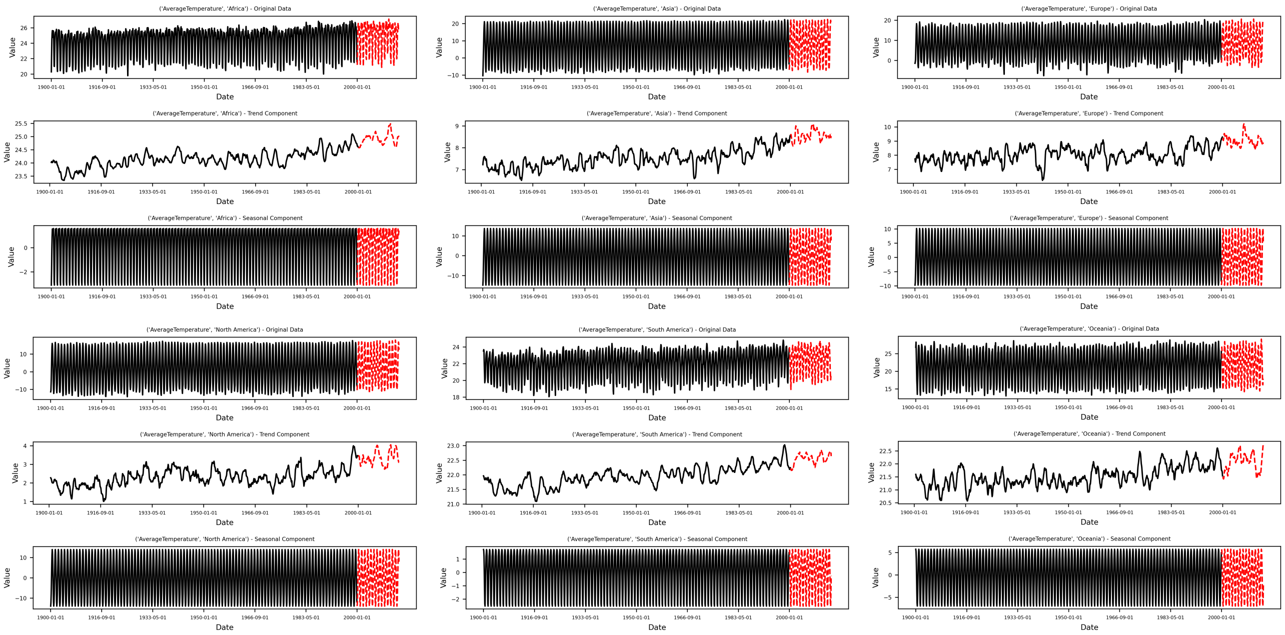

Supplementary Figure 8. Time series decomposition for variables from dataset DS 03 referring to continents.

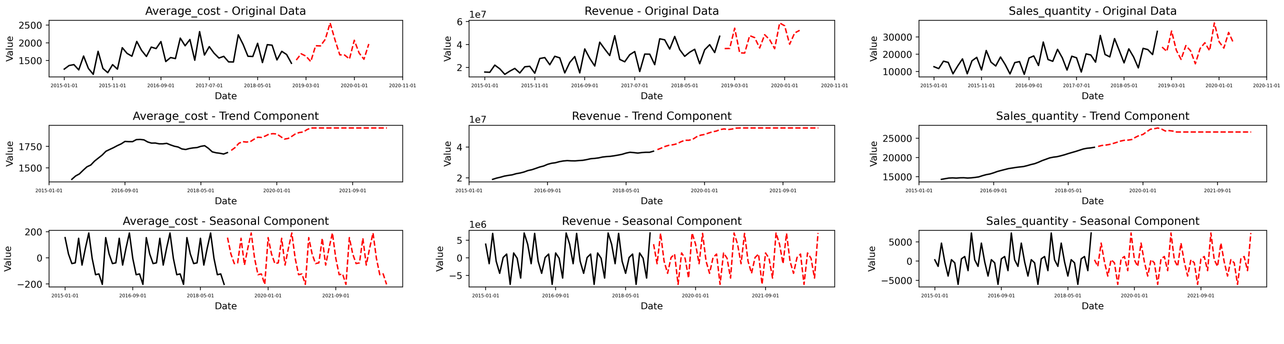

Supplementary Figure 9. Time series decomposition for variables from dataset DS 04.

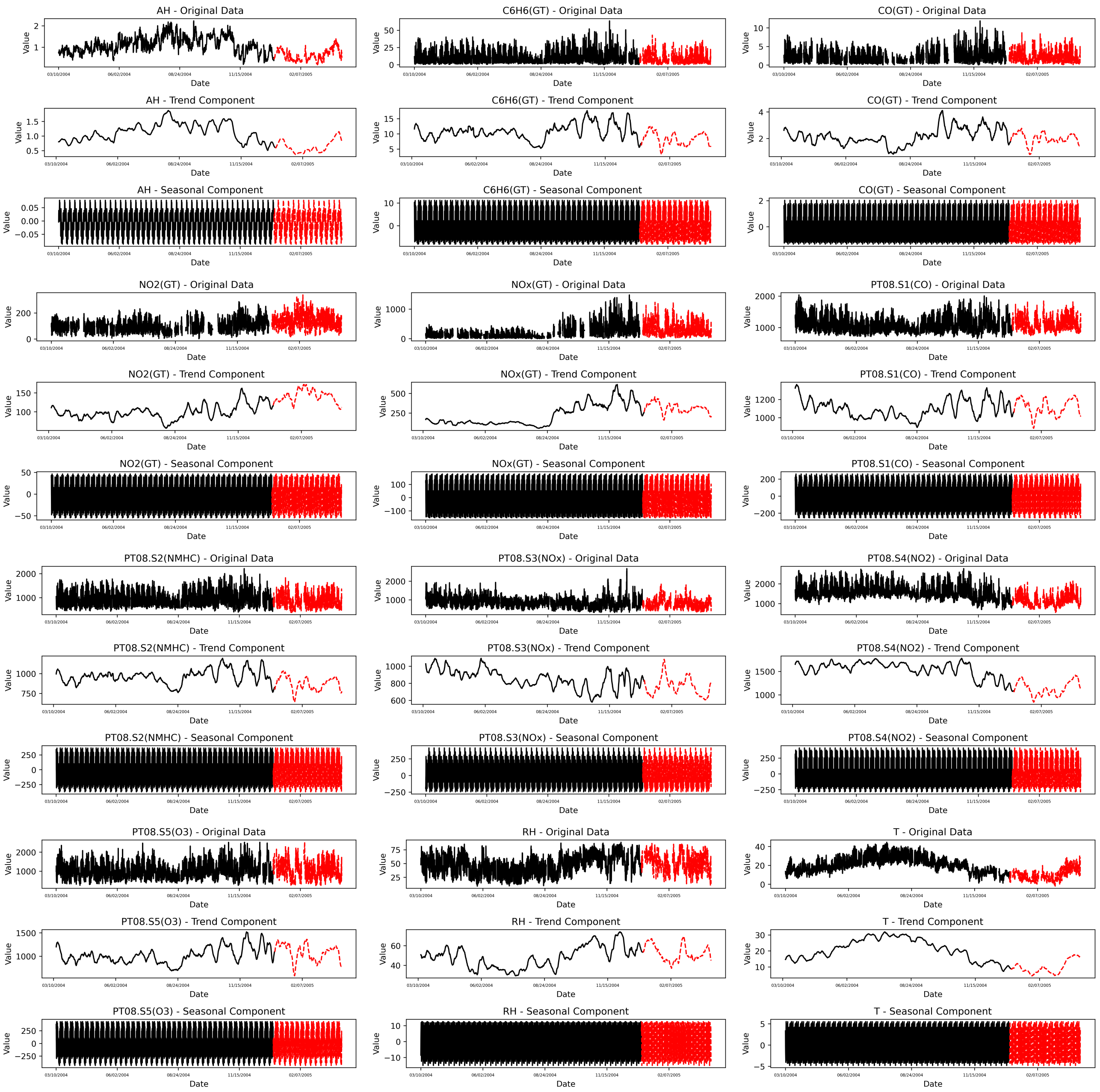

Supplementary Figure 10. Time series decomposition for variables from dataset DS 05.

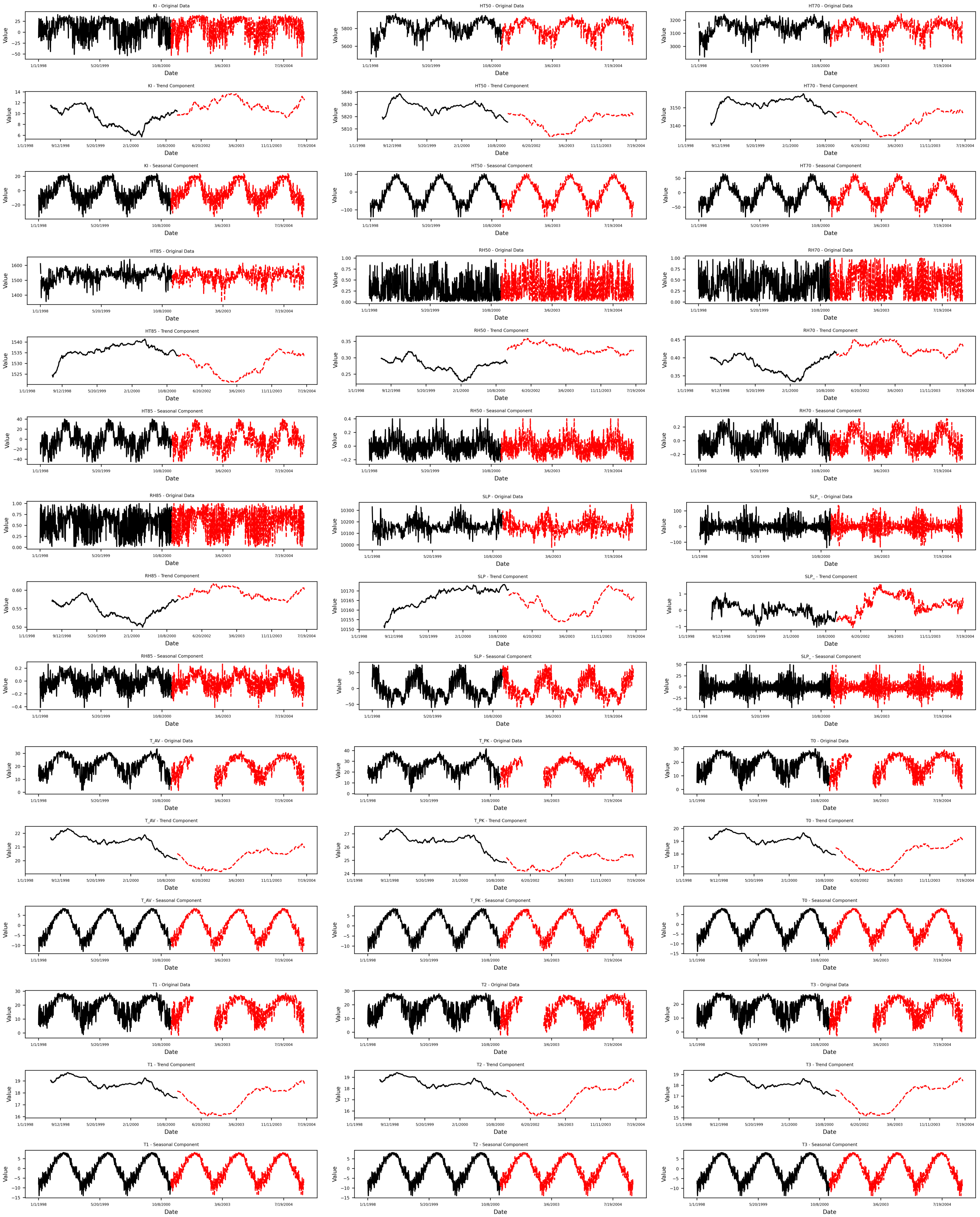

Supplementary Figure 11. Time series decomposition for variables from dataset DS 06 (KI to T3).

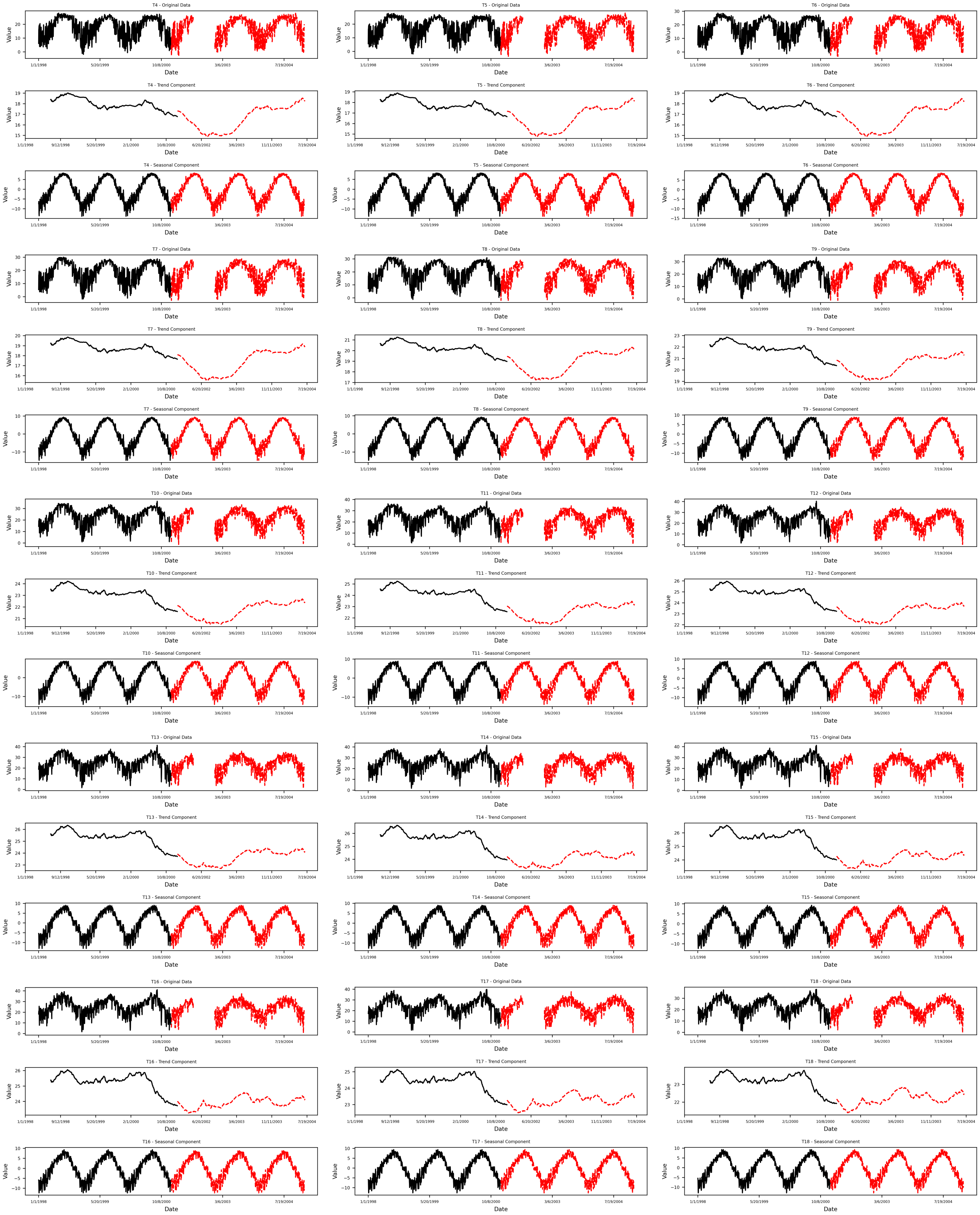

Supplementary Figure 12. Time series decomposition for variables from dataset DS 06 (T4 to T18).

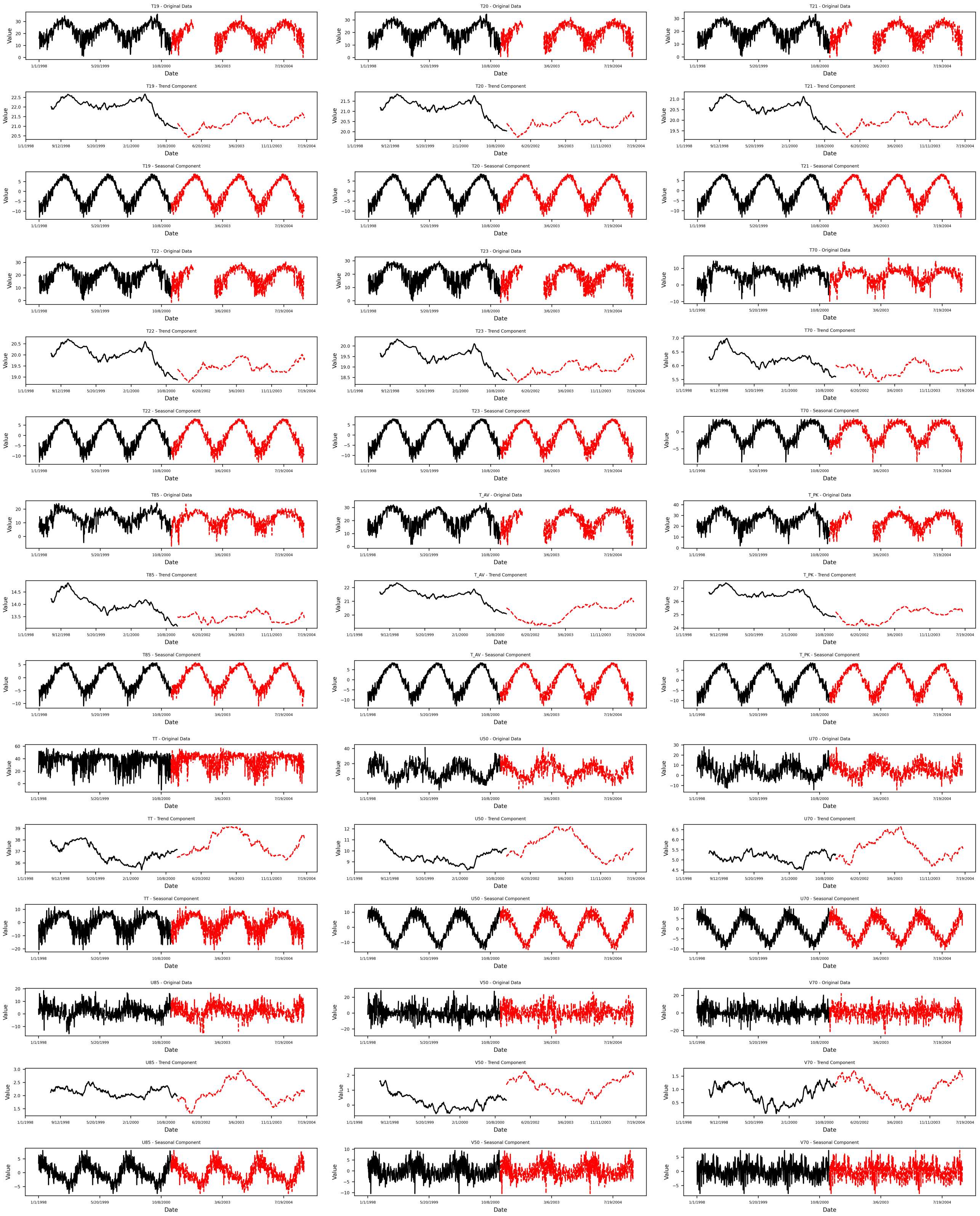

Supplementary Figure 13. Time series decomposition for variables from dataset DS 06 (T19 to V70).

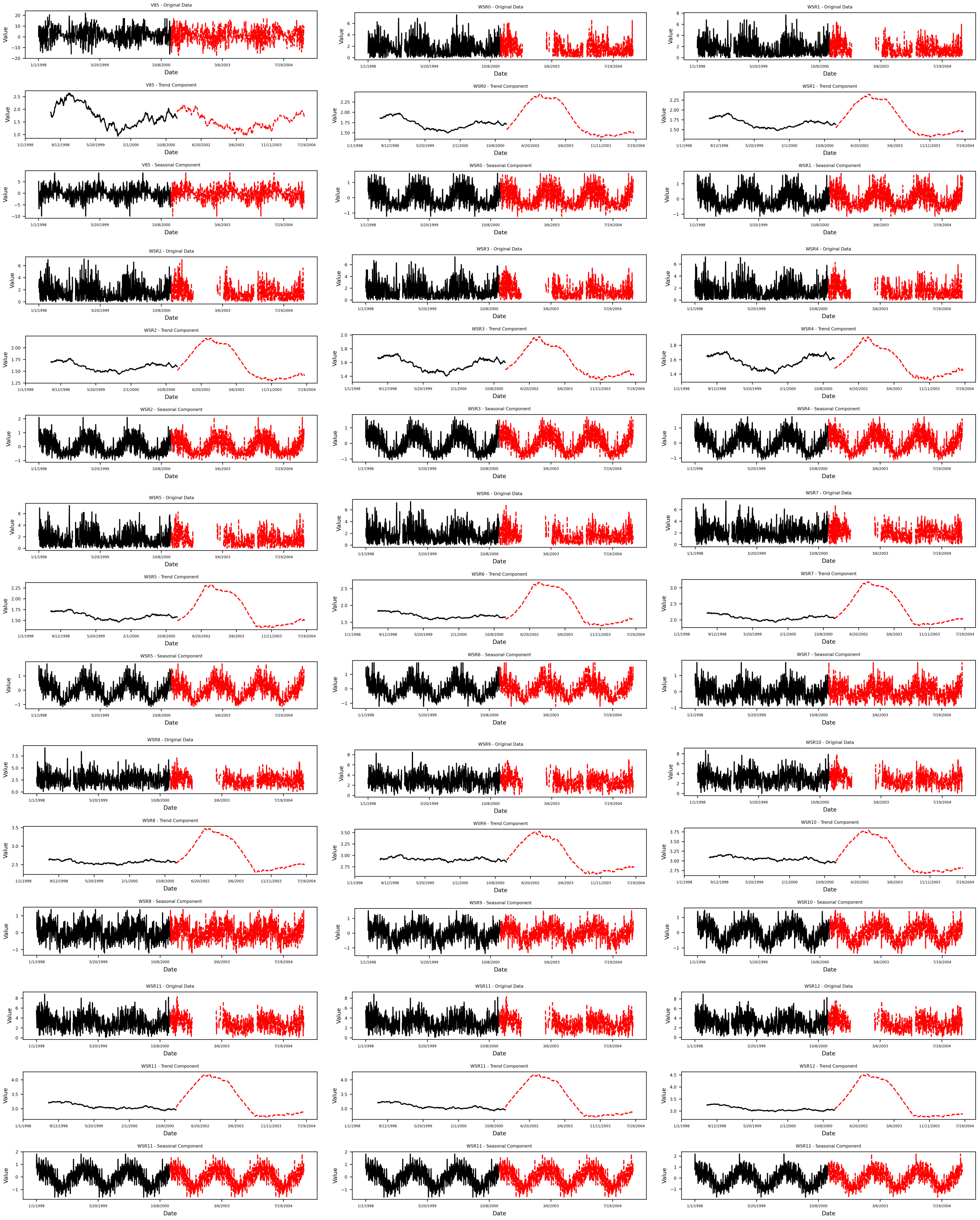

Supplementary Figure 14. Time series decomposition for variables from dataset DS 06 (V85 to WSR12).

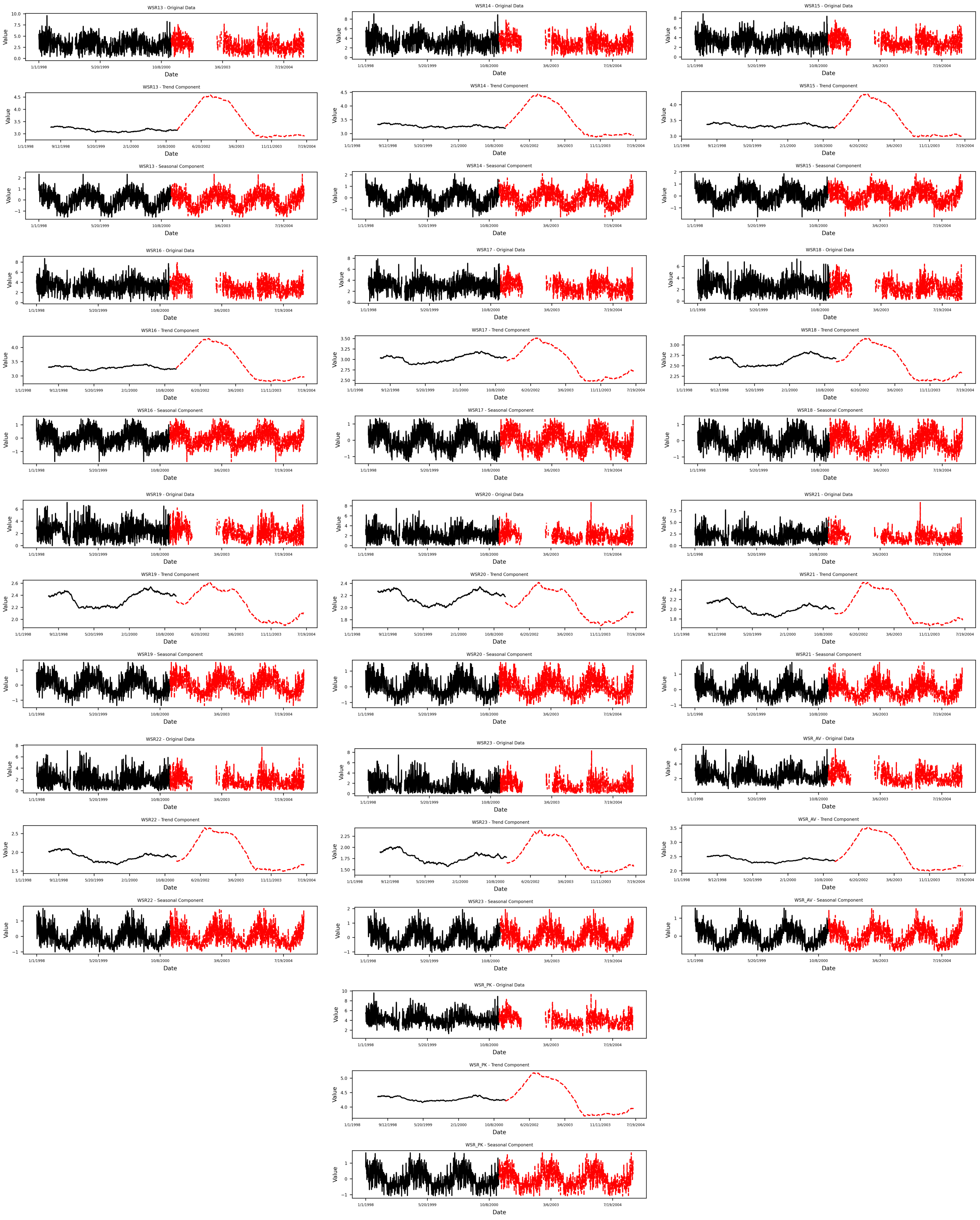

Supplementary Figure 15. Time series decomposition for variables from dataset DS 06 (from WSR13 to WSR_PK).

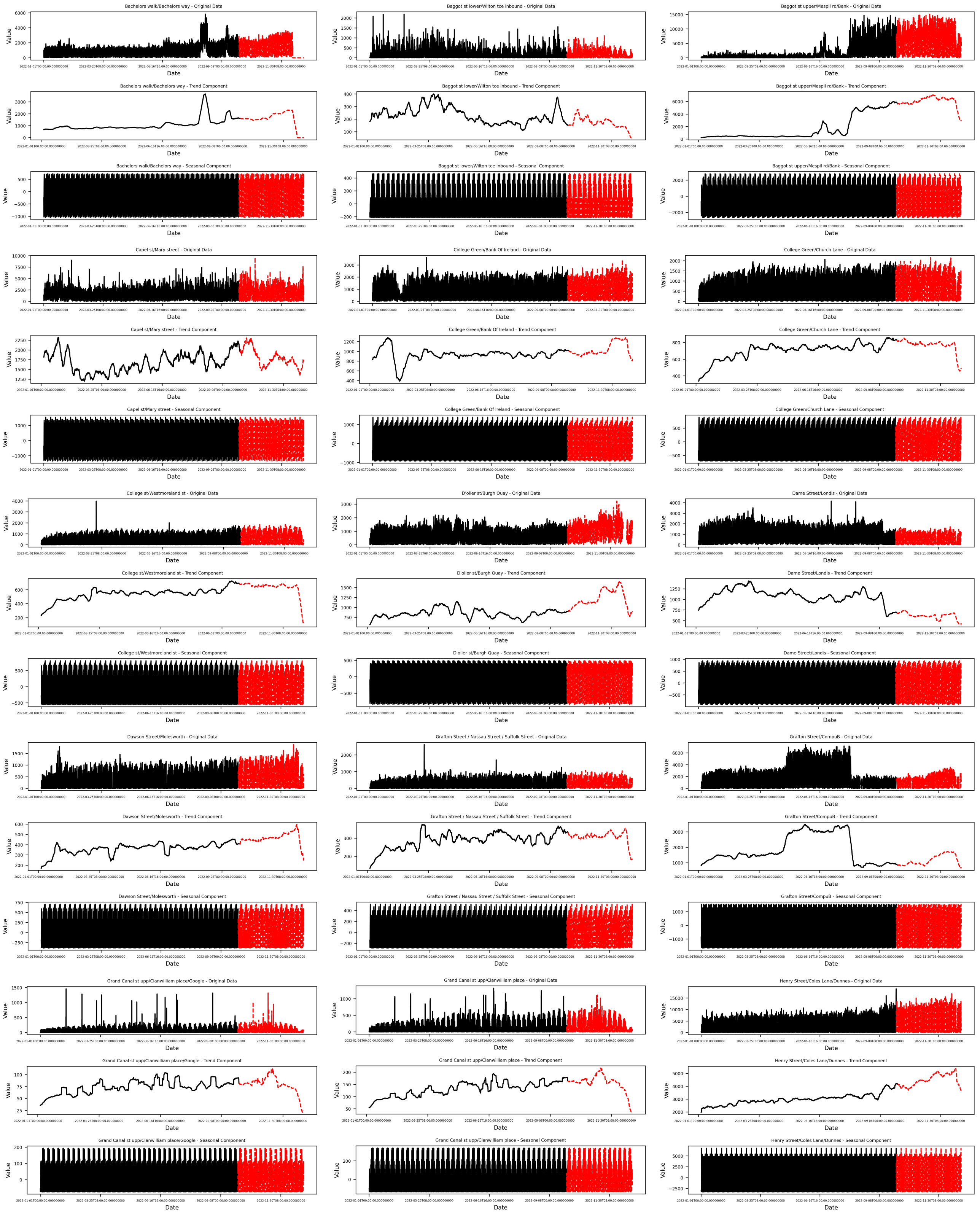

Supplementary Figure 16. Time series decomposition for variables from dataset DS 07 (from Bachelors' walk to Henry Street).

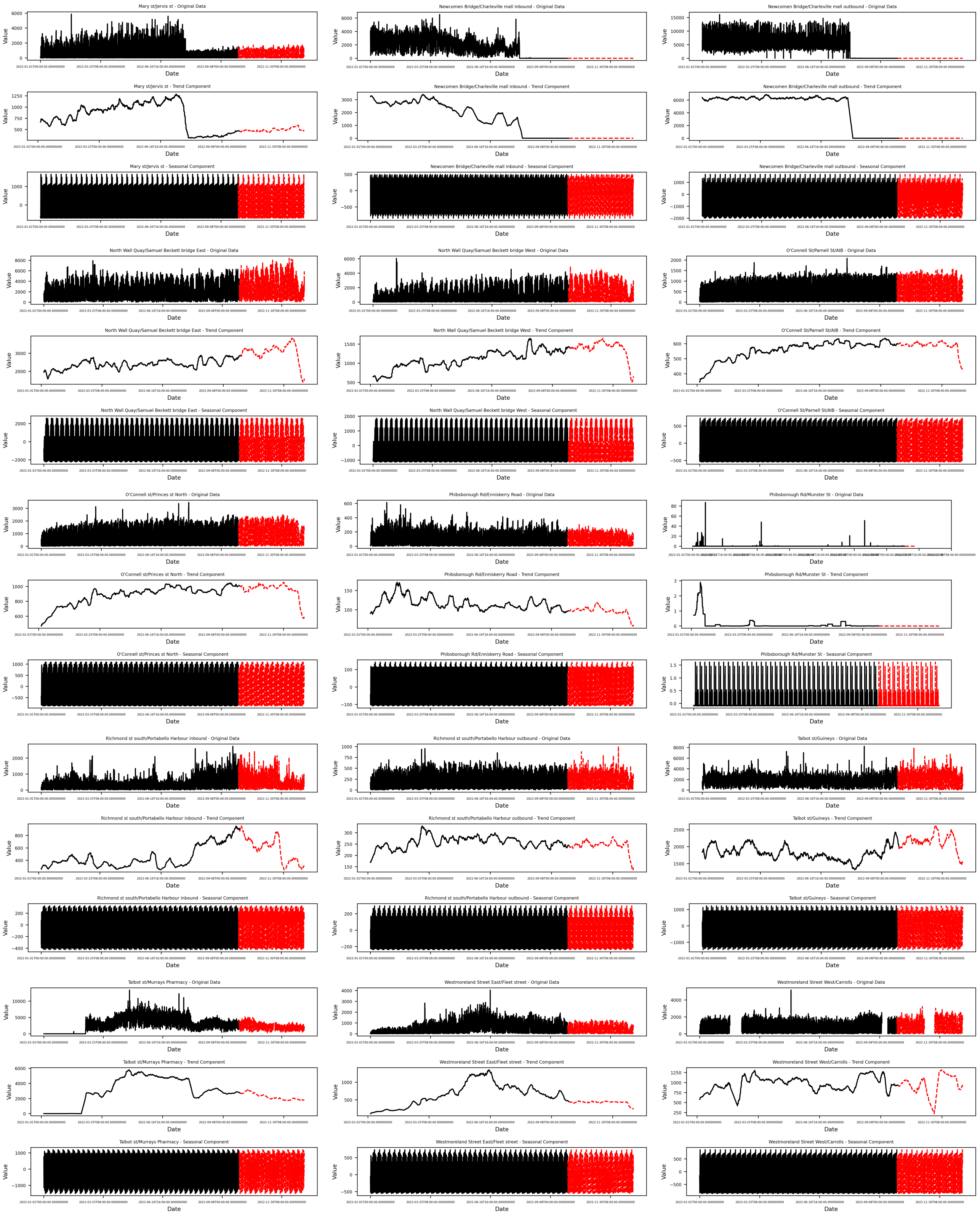

Supplementary Figure 17. Time series decomposition for variables from dataset DS 07 (from Mary Street to Westmoreland Street West-Carrolls).
